# Clinical Review, and Hospital Reports

**Published:** 1843-07-01

**Authors:** 


### Clinical Review.

---

### GUY'S HOSPITAL.

Guy's Hospital Reports. Second Series. No. I. April, 1843. Edited by George Hilaro Barlow, M.A. and M.D. Fellow of the Royal College of Physicians, and Assistant Physician to Guy's Hospital; Edward Cock, Assistant Surgeon to Guy's Hospital; Edmund L. Birkett, M.B. Secretary to the Clinical Society; J. H. Brown, and A. Poland. London: S. Highley, Fleet Street.

This number opens with an Introduction to the First Volume, which is not undeserving of a notice. The editors start with the observation :—

"If any opinion of the wants of the profession can be gleaned from the general spirit of its Members, and if any estimation of that spirit can be derived from the titles and subjects of publications, and the general tendency—at present so evident among us—to form societies for receiving and imparting the fruits of experience, we are fully justified in considering observation to be the governing principle of advancing medicine; and the 'Hoc age' of the present period to be, 'Ut vivas, vigila.' But it must be remembered, that however correct our facts, however trustworthy our modes of determining them, the progress of medicine must also depend on their comparison. The object, therefore, of any medical periodical, whose single aim is scientific improvement, should be twofold—the collection and publication of the results of observation—and the inferences which may thence be justly deduced. On this principle the present work has always been conducted; though the chief energy has been expended on fulfilling the second of these objects; viz. that of giving to the medical world the well-digested inferences of the result of observation in finished treatises. The course the 'Reports' have run, has not been unaccompanied with difficulty; and though perhaps the goal aimed at may not have been reached, still the experience of the seven years which have been now completed—and not, it may be hoped, without a certain amount of usefulness—may be received in evidence of the intentions of the editors, and the success which has been the result.

"We wish now to extend the sphere of this principle; and, in addition to the finished treatises, the works of individuals, with which hitherto our pages have been principally enriched, we propose to illustrate the different classes of disease by the aid of series of Reports, collected within the walls of the Hospital, and furnished by the books of the Clinical Society: and likewise, to apportion a part of each Number to the consideration of anomalous cases from the same source; —a plan we consider fraught with great advantage; as nothing has so great a tendency, either to counteract a too great confidence, or to stimulate exertion, as a proper estimate of our difficulties."

This is so obvious that it almost amounts to a truism. Medicine, like every science of observation, must rest upon facts, and those facts must necessarily form two orders—those which have an affinity to one another, and admit of being grouped and reduced into generalizations, and those which are of a more insulated character, and incapable of being so reduced, either from their own nature or the imperfection of our present knowledge, and so Reports must ever consist, as they always consisted, of matters of facts, generalizations, inferences upon the one hand, and of extraordinary, exceptional, anomalous instances upon the other.

The Editors proceed to give a sketch of the formation and development of the Clinical Society of Guy's Hospital. But this we may pass over. We cannot, however, do so, without expressing our cordial satisfaction at an institution so creditable to the officers of the hospital, who have fostered it, and to the pupils who are the members of it—an institution eminently calculated to breed a healthy spirit of observation in the young men whose zeal it stimulates, whose energies it directs, and whose prospects in after life it must beneficially influence.

The Editors proceed:—

"Their objects have been two-fold—the accuracy, and the number of the reports. And these objects they have essayed to accomplish, by facilitating the duties of reporting: 1st, By the division of labour: and 2dly, By the introduction of formulæ for case-taking. The labours of reporting are of course to be diminished by the number of cases under the charge of each reporter. The whole number of patients in the Hospital at one time is generally about 450—the number of Clerks about 30; giving, as a result, about 15 patients as the average complement of each clerk: and, calculating that there are weekly from 55 to 65 admissions, the duration of each patient's residence in the hospital would be about seven weeks. Each clerk would therefore have to report rather more than two fresh cases every week—a number, considered as an average, and consisting of mixed cases, both mild and severe, simple and complicated; evidently in the power of any student, at the requisite period of his studies, to do ample justice to, without neglecting his other subjects.

"The subject of formulæ requires a more lengthy notice. The objects to be aimed at, in their formation, are, facility of reporting with uniformity, and increased value of the reports. The first object has certainly been attained, even by the full formulæ now in use by the Society. Indeed many have been induced by them to commence a course of clinical study, the difficulties of which they confessed they should otherwise never have surmounted. So we may pass on to the second item; viz. uniformity and accuracy of the report. In the first place, if cases are to be compared—and such is confessedly one of the main objects of their accumulation—uniformity is almost essential; and in order to uniformity, formulæ are useful, if not positively necessary. These may be full or short, general or special. We believe the fuller they are, the greater is the amount of value accruing both to the reporter and the Society; although, as a point of direct consequence, it must be allowed that the errors would probably be more numerous than in shorter forms, though this only holds good as long as no stride is made beyond the defined limit. The next object contemplated is the fulness of the report, with the accuracy of its details; and for this purpose the formulæ must be full, containing a place for every possible item; and yet so arranged, that the reporter be not left in doubt under what head to note any particular symptom; and that space sufficient be allowed for each head. Special forms, although perhaps of great value in the reduction of matter from a large collected body of reports, imply—so at least it appears to us—a greater advancement in medicine that it has hitherto been our lot to enjoy; for how few diseases can be logically expressed—how widely diseases vary, both as to the number and intensity of the symptoms—and how little hope are we at present justified in entertaining that any law regarding the constancy of symptoms is about to be divulged—though we may perhaps expect some result from the consideration of their variations, and the endeavour to determine their value from their relations."

After remarking that the formulæ first introduced proved inefficient, and were of necessity abandoned, they continue:—

"With respect to the construction of formulæ, the rule appears to be concentration of that portion of the report which is usually so indefinite; viz. whatever precedes the admission of the patient, or the first visit of the physician;—with the expansion of that portion of which the reporter can institute his own investigation; viz. the enumeration of the signs and symptoms, suggesting to the physician the name of the disease; which may be usefully considered as a condensed arbitrary expression for the sum of the symptoms.

"The formulæ we at present employ were suggested by the short forms used by Dr. Conolly, at the Lunatic Asylum at Hanwell; and brought therefrom, in the course of the last summer, by the Honorary Secretary. They have of course been much modified; inasmuch as they are intended for diseases in general, whereas the others are specially designed for one class. They may be divided into several portions, as follows:—

"1. The relations of the patient with respect to the Hospital.

"2. The previous history of the patient.

"3. The causes of the disease, or the link between the previous history of the patient and the present history of the disease.

"4. The history of the present disease, with the date and the order of the succession of symptoms.

"5. The signs and symptoms of the disease, from the sum of which the diagnosis is to be determined.

"6. The history of the progress of the case with the treatment detailed.

"And these again are variously sub-divided, as may be observed by casting the eye over the plan given below.

No. Name of Disease Result

---

Ward

No. Physician, Date of Admission

Name Reporter

Age Single or Married No. of Children

Occupation

*Previous History.* { Abode

Habits of Life

General Health

Previous Diseases or Injuries }

*Causes or Influence* { Hereditary { Health of Family

Causes of Death in Family }

Moral

Physical }

History of Origin, with Date of Present Illness, and Order of Succession of Symptoms.

Development

*General Aspect.* { Natural

Morbid }

Position

Integuments and Appendages

Temperature

Moist or Dry

Colour

Œdema

Pain or Tenderness

Ulceration or Abscess

Tumors or Eruptions

State of Appendages

Nervous System

Form of Head or Spine

Pain

Vertigo

*Lesions of* { Intellect or Disposition

Motion

Sensation

Special Sensation }

Respiratory and Circulating System

Form of Chest

Digestive System

Form of Abdomen

| | |
|---|---|
| Pain | Pain |
| Voice | Appetite |
| Respiration | Nausea |
| Cough | Vomiting |
| Sputa | Tongue |
| Results of Percussion | Mouth and Throat |
| | Defæcation |
| Results of Auscultation | Dejections |
| Respiration | Results of Manipulation |
| | Urino-Genital System |
| Voice | Micturition |
| Heart's { Impulse | Urine |
| Heart's { Rhythm | Generative Functions |
| Heart's { Sounds | Lesions of Bones or Joints |
| Pulse and General Circulation | Treatment |

Progress of Case

| Treatment | Report |
|---|---|
| | |

We have inserted the whole of the preceding formulæ, because we think the subject an important one. We quite agree with the editors on the value, indeed the indispensable necessity for formulæ, in order to obtain reports on any scale or of extensive value. And we agree with them too, on the propriety of developing the present and tangible features of a case, while its history is placed in a subordinate position. For it requires little experience in case taking to learn how little dependence can be placed upon such history. When the defects of memory, habits of inaccuracy, and positive falsehoods on the part of the patient are added to the imperfect methods of examination, leading questions and possible bias on the part of the Reporter, the results are not such as constitute a sound basis for either practical or theoretical conclusions. And such we believe is found to be really the case.

To the formulæ themselves we have little to object. They are elaborate, and as complete as general formulæ can be. Some may think that they sin upon this side, but it must be recollected that they are for the purpose of systematic reporting, on the part of those who are to make it their business.

The contents of the number before us are as follows:—

Case of Suspected Irritant Poisoning, with Remarks on the Poisonous Properties of certain Kinds of Decayed Animal Matter used as Food; by Alfred S. Taylor.—Observations on Pelvic Tumors obstructing Parturition; with Cases; by John C. W. Lever, M.D.—An Inquiry into Certain of the Causes of Death after Injuries and Surgical Operations in London Hospitals, with a view to their Prevention; by Norman Chevers, M.D.—Observations on the Structure, Functions, and Diseases of the Coronary Arteries of the Heart; by Norman Chevers, M.D.—Observations on the Digestive Solution of the Œsophagus, and on the Distinct Properties of the Two Ends of the Stomach; by T. Wilkinson King: with a Case, by Mr. John Comley.—A Case of Glanders in the Human Subject; by H. M. Hughes, M.D.—Report of Cases of Hernia, admitted into Guy's Hospital from Sept. 1841 to Dec. 1842; by Alfred Poland, (with Plates.)—Account of Observations made under the Superintendance of Dr. Bright, on Patients whose Urine was Albuminous; by George Hilaro Barlow, M.D.; with a Chemical Examination of the Blood and Secretions, by G. O. Rees, M.D. (with Plates.)—Report of Cases of Fever; by J. H. Browne.

### I. Case of suspected Irritant Poisoning, with Remarks on the Poisonous Properties of certain Kinds of Decayed Animal Matter used as Food. By Alfred S. Taylor.

It appears that "three members of the family of a shepherd—the wife, son, and daughter, the two latter being young children—were taken ill on Sunday, December 20th. The boy, who was about two years old, died the following day. It was supposed that poison had been administered to the family, and that this was the cause of the boy's death.

"The poison was suspected to have been taken at dinner, about 11 A.M. on Monday, December 21st, when all three dined with the father, on some mutton. No satisfactory history could be obtained of the symptoms suffered by the wife and two children on the Sunday, the day preceding. The only account that could be obtained was, that the body of the deceased was swollen all over. The three were, however, better on the Monday. Having dined at the hour above-mentioned, and the father having left for his usual work, they were not seen until about two o'clock, when the mother and daughter were in a state of insensibility, and the boy was dead.

"The following account was obtained:—The father stated, that after he had dined with the family on the Monday at eleven o'clock, he felt, while at work, a sharp burning pain in his inside, for which he could not account. This was between the time of his leaving dinner and two o'clock in the afternoon. The mother, on her recovery, said that she felt great pain after the meal; but no other account of her symptoms could be procured, except that she foamed at the mouth, and was in a state of great nervous excitement. As far as could be ascertained, she had suffered but slightly from vomiting, and there was no purging. The deceased and his sister were, however, affected both with vomiting and purging. The deceased child died in *less than three hours* after the meal, for he was found quite dead at two o'clock; but no satisfactory account of his symptoms before death could be obtained. It appears, however, that he was very much purged, and that his motions were of a dark-green colour. The matters vomited by him were very copious, and streaked with a yellow-coloured substance: these were, unfortunately, thrown away. The matter vomited by the mother is described as having had a bright glistening appearance like quicksilver on the surface.

"The stomach-pump was applied to the mother about six hours after she was found (eight o'clock P.M.); and the contents of the stomach then drawn off were reserved for a chemical examination.

"The following appearances were met with, on a post-mortem examination of the body of the deceased child (reported by Mr. Cooke):—

"There was no particular appearance externally, except that the cutaneous surface was unusually pallid. The lungs were loaded with blood of a scarlet colour. The heart was natural: the liver of a pink colour, congested with very liquid blood. The stomach contained a small quantity of half-digested food, presenting, on its posterior part, several prominent rugæ, which were inflamed, with traces of inflammation on other parts of the lining membrane. The small intestines were inflamed in their upper portion; but the appearance of inflammation was less marked inferiorly. They contained a liquid mixed with blood. The muscular coat of the rectum was very red; but there was nothing in other respects abnormal, either in the large intestines or their contents. The peritoneum was highly inflamed. The bladder was contracted; and on its posterior wall were two spots of well-defined inflammation. The spleen and kidneys were healthy. About two ounces of bloody serum were found in the cavity of the abdomen. The upper part of the larynx and lower part of the pharynx were inflamed; and there were traces of inflammation at the bifurcation of the trachea. The veins of the head were very full, as well as those on the surface of the brain. The brain was of large size, and well developed: its vessels were congested; but there was no morbid change of structure."

No trace of poison could be detected in the contents of the stomach and viscera. The suspicion of poisoning, however, was entertained, and it was strengthened by the fact, that the man and his wife lived somewhat unhappily together. The husband, himself, who was a shepherd, kept by him a quantity of arsenic and bichloride of mercury, which he was in the habit of using for the purpose of destroying the fly in sheep. It was therefore suspected that one or other of these irritant poisons, or some preparation of barytes, had been administered by him designedly; or that the poison might have become accidentally mixed with the food, and have thus given rise to the illness of all the parties, and to the death of the child.

But there was another explanation of the symptoms. "The mutton which the family had had for dinner on the Monday was part of the body of a sheep which had been affected with 'the staggers,' and which, in consequence, had been killed, and the meat distributed among many poor families in the neighbourhood. It was therefore not unreasonably considered that the very unwholesome nature of this food might sufficiently account for the serious consequences which had followed the meal. It is however worthy of remark, that no other persons of other families, who had freely partaken of the mutton from this sheep, were attacked, or experienced any ill consequences whatever."

The meat could not be procured for examination, but a tin plate which covered it presented no poisonous contamination.

A medical opinion was required of Mr. Taylor, on the cause of death in the child. It might be ascribed to three causes.

1. To some mineral irritant poison mixed with the food.

2. To the poisonous nature of the food itself; either from the animal having been killed while in a diseased state, or from the flesh having become partially decayed.

3. To natural causes.

These hypothetical cases are examined with much ability by Mr. Taylor. The curious we must refer to the original. The pros and cons under each head are carefully weighed, and Mr. Taylor comes to the conclusion that there is no evidence of there having been a mineral poison at work—nor of death from natural causes—but that the meat was at the bottom of the mischief. He conceives that there are only three ways in which the features of the case can be explained.

(1.) That the effects were due to idiosyncrasy; the diseased mutton not possessing any injurious properties, but being rendered poisonous by peculiarity of constitution in this family.

(2.) Admitting that there was no such peculiarity of constitution, that the disease in the sheep had especially affected and rendered poisonous that particular portion of the flesh which had been taken by this family.

(3.) That the effects did not depend on idiosyncrasy, or on the disease with which the sheep was affected; but that decay had commenced in the portion of it assigned to this family, and that thereby an animal irritant poison had become generated.

Of these hypotheses, he is inclined to adopt the third; and, in the course of the paper, he cites some striking facts, in support of this opinion. One or two may not be out of place even here.

"At a public festival at Zurich, in the year 1839, upwards of 600 persons partook together of a repast, consisting chiefly of veal, roasted or in cutlets. At *variable periods* afterwards, nearly all of these individuals were taken ill; and in a week most of them were confined to their beds. They were affected with rigors, vertigo, headache, intense fever, diarrhœa, vomiting, and, in some instances, delirium. At a later period, an abundant flow of fetid saliva occurred, the interior of the mouth being covered with ulcers; and in many cases, after involuntary discharges of the fæces, great prostration of strength, and death, ensued. In these cases, the mucous membrane of the digestive canal was found softened, the intestinal follicles ulcerated, and the veins empty. It was afterwards ascertained that the veal, when eaten, had been in an *incipient* state of putrefaction."

In another case related by Dr. Christison, the putrefaction of veal had gone farther, so far indeed as to have converted it into something approaching to adipocere. Several persons who ate of it were all seized with pain in the stomach, efforts to vomit, purging and lividity of the face, succeeded by a soporose state, like the stupor caused by opium, except that when roused the patients had a peculiar wild expression. One patient died comatose in the course of six hours. The rest, being freely purged and made to vomit, eventually got well; but for some days they required the most powerful stimulants, to counteract the exhaustion and collapse which followed the sopor.

After partaking of a roebuck, killed in a state of excessive terror and exhaustion, several persons experienced a violent gastro-intestinal inflammation, with other symptoms like those already detailed. Here the flesh was *not* in a putrefying state.

But we need not pursue this subject. We strongly recommend our readers to peruse the original paper, which, like all of Mr. Taylor's on matters connected with medical jurisprudence, is distinguished by sound sense and correct reasoning.

### II. Observations on Pelvic Tumors obstructing Parturition; with Cases. By John C. W. Lever, M.D.

In a former paper, Dr. Lever made some remarks on those tumors which implicate the pelvis itself, as well as those organs and structures concerned in the birth of the child. He now turns to those tumors which belong to or implicate the parts in the neighbourhood of the birth passages. This division includes—*A.* Tumors of the ovaries. *B.* Tumors of the Fallopian tubes. *C.* Tumors of the rectum. *D.* Tumors of the bladder. *E.* Tumors in the cellular tissue of the pelvis: and, *F.* Those varieties of pelvic hernia which may and do occasionally offer an obstruction to the course of natural parturition.

### A. *Tumors of the Ovaries.*

The ovaries are liable to various diseases and displacements: thus, there may be inflammation and its consequences; there may be encysted dropsy; the ovary is liable to be affected with specific and malignant tumors; and lastly, it may be displaced or dislocated. Inflammation of the ovary may be either acute or chronic; and may cause induration, softening, or suppuration of the organ. The ovary is also liable to the encysted form of dropsy—to osseous, cartilaginous, and scrofulous and tuberculous tumors—to cancer and melanosis.

The ovary is subject to displacement within or without the pelvis. This may be a consequence of displacement of the uterus. Inflammation may fix the ovary in its morbid position.

The ovary may become displaced, when it may remain within the pelvic cavity, or escape from it. Displacement of the uterus may disturb the ovary; inflammation supervening on its malposition may glue it in its new place. The enlarged ovary falling into the recto-vaginal pouch may form a serious obstacle to the progress of labour.

In extra-pelvic displacement, the ovary may be found in an umbilical, ischiatic, femoral, or inguinal hernia.

"When pregnancy is complicated with enlargement or misplacement of the ovary, it is of great importance to ascertain the extent, situation, size, shape, density, and connexions of the obstruction. The enlarged ovary may be seated above the brim of the pelvis, or it may descend into that cavity occupying the side corresponding to the ovary affected: in other cases, although the ovary descends into the pelvic cavity, its mobility is so great, that there is no difficulty in lifting it up, and in placing it above the brim, as was done in Cases 34 and 35.

"Where the tumor is of the congested form, large, and confined to the abdomen, it may produce lateral obliquity of the uterus; and so render the labour tedious, preventing the presentation from entering into the brim, as in Case 34."

Ovarian tumors, complicated with pregnancy, vary, as might be supposed, very much in size. They have been known to occupy the whole pelvic cavity.

It is of consequence to ascertain the consistence of these tumors. If fluctuation is indistinct, Dr. L. approves of an exploratory puncture. In short, he is of opinion, that, in all tumors of this kind, impeding labour, we are justified in assuring ourselves of the nature of the tumor before proceeding to perform the operation of embryotomy.

The diagnosis of ovarian tumor, when complicated with labour, is not always easy, particularly when the patient is not seen till she is in labour.

"In forming our diagnosis, we must be guided by the history, situation, and shape of the tumor. If an examination be made before the child's head has descended into the pelvis, or if there be no adhesions and the tumor be moderate in size, its displacement and reposition may enable us to determine its nature. If the tumor be encysted, it is elastic, soft, and fluctuating, becoming more tense during the uterine efforts; or by them it is urged into the pelvic cavity, or the child's head is pressed against it, which took place in Case 38. But otherwise, whether it be of a non-malignant or malignant nature, uterine pains will not increase its tensity, neither will it become flaccid when the efforts cease; in short, the only effect produced by the pain is a forcing down of the tumor *en masse;* so that, in order to arrive at a correct diagnosis, we should examine the tumor both during and in the absence of pains. Exploration already referred to should be made in every case in which doubt exists, before we proceed to destroy or even risk the child's life. The history of the examination of the case, both *per vaginam* and *per anum,* will, I think, in every case, enable us to decide the obstruction to be ovarian; for if due caution be employed, it can neither be mistaken for disease of the uterus nor vagina. The cases which are most likely to be confounded with ovarian tumors are those in which encysted or other tumors are developed in the cellular tissue, between the rectum and the vagina; but this is but of little consequence, as the treatment in both cases is founded upon the same principles."

*Prognosis.*—This must be considered in reference to the mother, and the child. It must mainly depend upon the size, seat, mobility, and nature of the obstruction.

"If the tumor be small, if it chiefly consist of fluid, or if we are able to return it above the brim of the pelvis, there is little or no danger to be apprehended, either to the mother or the child; but if the tumor be large, solid, firmly adherent, and if it occupy the cavity of the pelvis, the operation of craniotomy will very probably be necessary; and the difficulty in performing that operation, as well as the injury the parts may sustain during the delivery, will render the recovery of the mother a question of great uncertainty: or, lastly, the labour may be so protracted, that the patient may die of exhaustion. Puchelt, who has collected together thirty-one cases, mentions but one (that related by Busc) in which the woman died undelivered, although he alludes to several which proved fatal after delivery, either from exhaustion or inflammation. Our prognosis, therefore, must be based upon the nature, size, and mobility of the obstructing tumor. Of the thirty-one cases related by Puchelt,

1 died without being delivered;
14 died soon after delivery;
3 died from other causes; and
13 recovered.
—
31

Dr. Merriman states that, out of eighteen cases,

9 women died:
3 ...... recovered imperfectly;
while 6 ...... recovered perfectly.
—
18

With regard to the child, our prognosis will depend on the same causes that influence us in determining that of the mother.

Puchelt states, that—

21 children died before delivery;
2 ........... after delivery;
7 ....... were born alive;
while in 2, the result is not stated.

In Dr. Merriman's cases, the still-born children amounted to 16, and those born alive to 4."

*Treatment.*—If the tumor, from its site, offers little obstruction to the birth of the child, or if it be so compressible as to let the head pass, labour may be naturally completed. Sometimes, where the tumor is caused by encysted dropsy, it is quite above the pelvis, and, so soon as the head is engaged in the pelvic cavity, the labour goes on naturally: but owing to the obliquity of the uterus caused by the tumor, the first stage of labour is lingering, as the head, or presenting part of the child, does not enter the brim of the pelvis in the most favourable manner. In all cases, whether the tumor is above the pelvis or within it, we should give full trial to the natural efforts; at the same time taking care not to defer our assistance too long, as the injury the soft parts may sustain may lead to fatal results.

*Reposition.*—In some cases, the tumor may be placed above the brim of the pelvis, out of harm's way, until the head has entered the pelvic cavity, when no obstruction is then to be apprehended. All accoucheurs concur in recommending this, when it can be effected, and there are many recorded instances of success under great difficulties.

*Puncture or Incision.*—" Dr. Merriman states, that in six cases, out of those recorded by him, the tumors were opened; in three, the labour was left to Nature to complete. Two of the women recovered, but the other remained for a long time in an ill state of health: two of the children were preserved. In the remaining three cases in which the tumors were opened, the use of the perforator was afterwards necessary: one of the women died, one remained in an ill state of health for eighteen months and then sank under her sufferings, while the third recovered. Dr. Ingleby is an advocate for the operation; and the case recorded at p. 126, which I have previously referred to, shews how likely even experienced medical men are to be deceived by the mere feel of the tumor. In this case, the sensation conveyed to the finger was that of an osteo-sarcomatous tumor: it felt precisely like a large mass of cartilage: it was not softer in one part than another: there was no fluctuation; and it was not harder during the pains than during their absence; but still, when a long curved trocar was introduced into the tumor through the rectum, about seven ounces of a clear straw-coloured viscid fluid, which proved to be albuminous, escaped through the canal: and although this was not followed by the natural expulsion of the child's head, still it rendered the operation of artificial delivery much more easy: and in Case 36, the tumor was supposed to be a solid body. I have before stated that, in my opinion, in every case, we ought to perform the operation of puncture before we have recourse to the more serious operation of embryotomy."

If the contents of the tumor are too thick to pass through a canula, an incision may be made. Some advise this to be made per rectum—others per vaginam. To this latter more natural opinion, Dr. Lever inclines.

*Extirpation of the Tumor.*—The following are Dr. Lever's sentiments on this head.

"If the tumor be of such a size or nature that its contents may be evacuated or lessened by the operation of puncture or incision, the operation of extirpation should not be entertained; neither, in my opinion, are we justified in resorting to extirpation, if we can deliver the child with the aid of the embryospastic instruments. But if they are inadmissible—if the tumor be so large, firm, and adherent, that, by its bulk, it prevents the descent of the child's head—by its incompressibility, it does not yield to pressure—by reason of its solidity, its contents cannot be discharged, either by the operation of puncture or incision—and by its confined and fixed position in the pelvis, it cannot be replaced—then I am of opinion that the operation for extirpation is called for, and justifiable."

He cites Dr. Merriman in support of this opinion.

*Turning.*—This Dr. Lever condemns. For, he argues, "if the tumor be so compressed that the hand can be readily introduced for this purpose, it is far better to wait, and trust to the natural efforts, or to evacuate the fluid contents of the obstructing tumor: for the operation of version must be attended with some degree of violence and risk; and indeed, should we succeed in passing our hand and bringing down the feet, the greatest difficulty will be experienced in the delivery of the shoulders and head. In five cases related by Dr. Merriman, the labour was terminated by turning the child: all the children were lost, and but one of the mothers recovered."

*Delivery by Embryospastic Instruments.*—Dr. Lever conceives that these cases are not generally suited for this mode of delivery. If instruments are required, they are usually those which evacuate the tumor, or remove at once, or those which lessen the size of the child and reduce it to the size of the contracted opening through which it has to pass.

*Cephalotomy.*—"Six cases are recorded by Puchelt in which the operation of cephalotomy has been had recourse to. Of these six, but three women recovered. Dr. Merriman states, that in his eighteen cases 'the perforator was used five times, after a longer or shorter duration of labour.' Of these women, three died; another recovered very imperfectly; and one got well. The perforator was had recourse to also in three cases after the tumor had been opened, one of these women died, one remained in an ill state of health for eighteen months and then sank under her sufferings, while the third recovered."

*Cæsarean Section.*—Dr. Lever knows of no case in which this has been performed.

*Induction of Premature Labour.*—After some remarks, Dr. Lever concludes, that, if we find the tumor solid, firmly fixed in the pelvis, and not permitting reposition—if from our examination we are convinced of the impracticability of using the embryospastic instruments—then the operation for the induction of premature labour may be had recourse to.

Dr. L. relates six cases, for which we must refer our readers to the Reports themselves. We proceed therefore, to

B. *Tumors of the Fallopian Tube, obstructing Parturition.*

The conditions of the Fallopian tubes which may lead to the protraction of labour, are abscess, the result of acute inflammation, dropsy, and tumors, both of the non-malignant, and malignant form. Cases of this kind occur but seldom. He quotes one which occurred to Chambry de Boulage. He goes on to observe: "When the tube is affected with swellings or tumors, it is found to leave its natural situation, and to occupy the pelvis below the pubic bones, but inclining more or less to the side where the tumor grows. If such a case should present itself to the accoucheur, the tumor should, if possible, be placed above the brim of the pelvis; but if this be impossible, it may be punctured, to permit the discharge of its contents: but if they be of so tenacious and thick a nature that they will not flow through a canula, then an incision may be made, in order to empty the containing sac. But if the tumor should be solid, and the pelvis seriously obstructed, we shall have to decide between the performance of the operation of cephalotomy and Cæsarean section. During the performance of the latter, we may possibly be able at the same time to remove the obstructing tumor."

C. *Tumors of the Rectum obstructing Parturition.*

1. *Tumors of the Rectum from Impacted Fæces.*—Such a cause of obstructed parturition is a well known one. The examination *per rectum* confirms what that *per vaginam* indicates. The double examination is obviously requisite. This source of obstruction can only occur when patients have not been sufficiently attentive to their bowels during the latter months of pregnancy. Glysters, or the scoop, and purgative medicine are the remedies.

2. *Scirrhus.*—Dr. Lever gives a case which we need not introduce. Examination per rectum et vaginam, determines the nature of the malady. Its *treatment* quoad delivery, is thus set forth:—If labour be interfered with, he thinks that the forceps will, in most cases, be sufficient to complete the delivery: at the same time, when we employ them, we must remember that, generally, the recto-vaginal septum is more or less implicated; so that it does not admit of that distention which, in its normal condition, it will allow. But if these be employed, and if, after gentle and judicious traction, they are found unequal to accomplish the delivery, we have then to decide between the delivery of the child by the operation of cephalotomy, and the performance of the Cæsarean operation.

D. *Tumors of the Bladder impeding Parturition.*

1. *Distention of the Bladder.*—This may obstruct labour, not only by interfering with the action of the womb, but by preventing the child from occupying the cavity of the pelvis.

The retention of urine is occasioned by the pressure of the presenting child on the neck of the bladder or urethra. Nor is it necessary that the full period of gestation should be accomplished for this stoppage to occur. M. Costes relates a case in which it happened at seven months and a half. The catheter could not be passed, and it was necessary to puncture the bladder. Rupture of the viscus has been known to take place. The bladder may become much dilated, so much so as to contain eleven pints of urine.

In some cases, the urethra is elongated, so that, although a full-sized catheter be passed, it is not sufficiently long to reach the chamber in which the urine is confined, or the urethra may be blocked up by a small calculus. Paralysis of the bladder may exist. Whatever be the cause, it is of the first consequence that distention of the bladder be not overlooked.

"When such cases present themselves to our notice, we must not be satisfied with the reports of nurses; for it is no unusual thing for them to say, 'the water is constantly dribbling,' or, 'flowing' even when the obstruction is so complete, that none, by any possibility, could have escaped. Medical men themselves occasionally fall into this error. I have, on more than one occasion, found the great distention of the bladder to be the obstructing cause of labour, when the surgeon in attendance has reported the passage of the urine to be copious and free. When the bladder is distended, pressure by the hand causes much suffering; and every return of labour-pains, accompanied as they are by contraction of the abdominal muscles, greatly augments the sufferings of the patient, from the pressure upon the distended viscus: in fact, in most cases, the woman loses all other pains, and complains of those seated at the bottom of the belly. When we examine to ascertain the state of the os uteri, we oftentimes find it inclined backwards; and if we succeed in pushing up the uterus to ever so slight a degree, a gush of urine will take place, but only in those cases where the distention is caused by the pressure of the presenting child. Moderate attention on the part of the accoucheur will, in most cases, suffice to prevent the accumulation of the urine; for if the patient be unable to pass the water, the catheter should be from time to time introduced. But if we are called to a case in which there is distention, we should first pass a common-sized flat female catheter. If there be a calculus at the commencement of the urethra, opposing the introduction of the instrument, we should endeavour to extract it by means of a pair of forceps: and if we should not succeed with them, the calculus, if of small size and not likely to interfere with the subsequent stages of labour, may be passed backwards into the bladder, and its removal left for a subsequent opportunity: but if its size be so large, that it alone may lead to the obstruction of the labour, or may cause sloughing or laceration of the neck of the bladder, we should remove it by an incision; as by this operation we not only get rid of the accumulation, but also of the cause of the accumulation; and thus a double obstruction is removed. If no calculus exist, and the common-sized catheter is found to be too short to reach the accumulation, an elastic gum male catheter, or one flattened like the female catheter, may be employed. In passing this instrument, it is generally necessary to raise the presenting part of the child with one or two fingers of the left hand, when the instrument is readily passed on, and the bladder emptied. If our best endeavours fail to pass either instrument, it will be better to puncture the bladder above the pubes, than run the risk of a rupture taking place, which will inevitably prove fatal."

2. *Calculi in the Bladder obstructing Labour.*—There are sufficient cases on record to prove that this mode of obstruction may occur. The diagnosis may not be very easy. Vaginal examination may lead to the suspicion—the history of the previous symptoms may strengthen it—but sounding alone can confirm it.

"I have stated, that the tumor formed by an obstructing calculus is usually moveable in the absence of pain; but it sometimes happens that the tumor becomes firmly wedged between the head of the child and the arch of the pubes, if the labour be allowed to go on without assistance. If the calculus, during the descent of the head, remain at the upper part of the vagina, it will not impede the delivery; and the head, descending into the pelvis, will itself prevent any future obstruction from the foreign body: but if the calculus fall into the neck of the bladder, and be placed below the head, the labour will be rendered difficult; since the conjugate diameter of the pelvis is contracted by the presence of the foreign body. In forming our prognosis of labour complicated with stone, we must take into consideration not only the size but the shape of the calculus; for the stone may be so large, that although it be seated in the bladder itself, and not interfere with the inlet of the pelvis, yet by its size it may, when pressed against the uterus by the contraction of the abdominal muscles, seriously interfere with the powers of expulsion; and, of course, if seated in the neck of the bladder, the amount of obstruction must be proportionate to the size of the stone: and even if labour be accomplished without the employment of instruments, the injury which the neck of the bladder may sustain will be followed either immediately or remotely by a vesico-vaginal fistula, which may embitter the remainder of the patient's days."

*Treatment.*—If the calculus is discovered during pregnancy, it should be removed before labour commences—if only detected when the patient is in labour, the case may be left to the natural efforts, if the calculus be small, or if it be seated in the cavity of the bladder above the head of the child. If the stone is large, or placed below the head of the child, other measures are requisite. The stone, if possible, should be pushed back into the cavity of the bladder, above the brim of the pelvis, by introducing a catheter into the urethra, and one or two fingers into the vagina. If the calculus cannot be replaced, it must be extracted, either through the urethra or by lithotomy above the pubes.

Extraction through the urethra by gradual, or more sudden dilatation, appears, says Dr. Lever, most applicable to those cases in which the calculus is seated at the commencement or in the urethra itself. Incontinence of urine not unfrequently follows.

If dilatation is inadmissible, lithotomy may be resorted to. It should not be deferred too long.

"The *high operation*, which has been recommended by some writers, appears to me to be more fitted for performance during gestation; as in the case related by Velpeau. The operation of version, advised by some authors, as Smellie, Velpeau, &c. appears to me to be entirely out of the question: for although we may introduce the hand without difficulty, and turn with great ease, yet there will be the same obstruction to the passage of the child's head which there was when the head itself was presenting. If the stone be of small size, and the head be engaged in the pelvic cavity, its advance may be assisted by means of the forceps. The perforator was resorted to in the case related by Threlfall; but the nature of the case was not properly understood by him when he undertook the performance of the operation; for he mistook the obstruction for one connected with the ovary, and did not even pass a catheter to ascertain the state of the bladder. But rather than have recourse to this operation, I think it will be much better to remove the stone in one of the modes above recommended: for, in my opinion, we are not justified in taking the child's life, when it can be saved by the performance of so easy an operation as that of lithotomy, which at once removes the obstruction, and does not subject the patient to greater risk than the operation of cephalotomy, under the circumstances."

3. *Obstruction to Labour, caused by Scirrhus of the Bladder.*—Dr. L. has seen one case of obstruction produced by a scirrhous condition of the bladder and anterior wall of the vagina. The disease will be recognized by vaginal examination, combined with the symptoms and general diathesis. Such a case must be treated like one of scirrhous rectum.

4. *Descent of the Bladder obstructing Labour.*—"The bladder in some cases descends before the child's head during labour, forming a serious obstruction to the process of delivery. It may descend during gestation; or it may take place for the first time during the early stages of labour, before the head is engaged in the pelvic cavity; and it probably is caused by the pressure exercised by the descending head upon the fundus or middle portion of the viscus, at a time when it is partially distended with urine.

"The symptoms which attend this displacement are, a bearing down in the region of the pelvis, accompanied by the sensation of pain, dulness, and soreness; there is a dragging from the umbilicus, or from a spot between it and the pubes: there is constant desire to pass the water; but the attempts to micturate are fruitless, although each return of uterine efforts is, in some cases, attended with the discharge of urine. When a vaginal examination is made, a tumor is found seated at the upper anterior and lateral part of the vagina, of a more or less oval form, smooth, fluctuating, and varying in size, according to the amount of displacement, and the quantity of water the bladder may contain. The tumor is seated below the head, if that present; and every successive return of uterine effort increases its tensity, especially in those cases where the urine does not escape. The size of these tumors varies: in some cases they are as large as the head of a new-born child. If a catheter be introduced into the urethra, it will at once detect the seat and nature of the tumor, and relieve the bladder of the accumulation, and so remove the obstruction."

The prolapsed bladder has been mistaken for a hydrocephalic head, for the membranes of the ovum, and punctured in consequence—or it may be confounded with ovarian tumor, encysted tumors of the vagina or pelvis, and vaginal hernia. The evacuation of the swelling by the catheter marks the diagnosis.

The treatment consists in the continual introduction of the catheter into the bladder, and the evacuation of the urine until the head of the child occupies the pelvic cavity, and prevents both the accumulation and descent. After the evacuation of the urine, the bladder should be passed up, and retained in its situation by two fingers of the left hand until the head occupies the pelvic cavity.

The perineum is in some rare instances the seat of a vesical hernia.

E. *Tumors of the Cellular Tissue of the Pelvis, obstructing Labour.*

The tumors of the cellular tissue of the pelvis have been divided into steatomatous, scirrhous, encysted, and hydatic tumors, to which may be added such as are occasioned by pelvic abscess.

1. *Steatomatous Tumors* may arise from any part of the pelvic cavity: they have been found growing from the sacro-sciatic ligaments, from the cellular tissue between the vagina and urethra, and from that covering the linea innominata, as well as from that lining the sacrum. The size of these tumors varies: in some cases they occupy almost the whole of the pelvic cavity.

2. *Scirrhous Tumors*, as scirrhous glands, may be situated along the cavity of the sacrum, and "may be recognized by their locality, irregularity, and hardness. Examination of these swellings usually causes much pain; and they are found to be placed externally to the vaginal coats, and more or less firmly attached to the surrounding structures. This condition of the several glands is usually associated with cancer of the womb in an advanced stage; and although the os uteri may have permitted the head to pass through." Dr. L. relates a case in point.

3. *Encysted Tumors.*—"These may be generally recognized by their fluctuation, by their being capable of being defined, by their freedom from pain, &c. They may readily be distinguished from hernia of the bladder or rectum, for which at first they are likely to be mistaken: the introduction of the catheter into the bladder in the one case, and the finger into the rectum in the other, will at once determine the point: but they are not so easily distinguished from dropsical or enlarged ovarium; neither is this of much importance, for the same principles which guide us in the management of the one case will direct us in the treatment of the other."

4. *Hydatids.*—A well-marked case is related by Meyer.

5. *Pelvic Abscess.*—This is preceded or attended by the ordinary symptoms of the formation of matter. It may be mistaken for an enlarged and dropsical ovary. A puncture suits both.

*Treatment.*—It is not only necessary to ascertain the situation, size, and boundaries of these tumors, but their solidity and firmness also. "If the pelvis be of good size, the labour will most probably be terminated by the natural efforts; as in the case related by Dr. Denman, in his Second Volume. If the natural efforts are unequal to the delivery, I do not think we shall save the child's life, or in many instances succeed by the use of the forceps. Dr. Blundell says, 'The application of the forceps in these cases is an excellent topic of obstetric dispute;' but unless the tumor is very small, we may scarcely hope to accomplish the delivery by the use of this instrument: in short, the attempt at its introduction in some of the cases would have failed, whilst in others, if applied, its use would most probably have been disastrous. In the majority of these cases, therefore, I am of opinion that the forceps are inapplicable." "The attempt at reposition will not succeed in most of these tumors; for they are generally firmly attached to the parts in which they are imbedded, and therefore will not admit of removal, from the pressure of the presenting child. Version, recommended by some authors, I regard as entirely out of the question; for by altering the position of the child we do not remove or lessen the opposing cause; we merely afford the accoucheur an opportunity of using more violence in attempting the delivery, and thereby sacrificing the life of the child, and endangering that of the mother. In the case of Mrs. S., related by Dr. Merriman in the Tenth Volume of the Medico-Chirurgical Society's Transactions, where the operation of turning was performed, the lives of both mother and child were lost. In a case related by Osiander, where the tumor grew from the middle of the left linea innominata, the child was still-born, although the mother recovered. If the tumors be of a fluid nature, as encysted tumors or abscesses, they should be punctured, in order that their contents may be got rid of; but if the substance contained within the cyst be too thick to pass through a canula, an incision should be made, in order that the sac may be emptied; when, the cause of obstruction being removed, the delivery of the child may be left to Nature, if the uterine efforts still continue; but if they have ceased, its extraction may be accomplished by means of the embryospastic instruments. If the tumor be so large as to occupy a considerable portion of the pelvis—if it be so dense and solid, that an exploratory needle passed into it fails to detect any fluid—if it be thought of a scirrhous or steatomatous nature—then we have to decide between the removal of the tumor, the performance of cephalotomy, and the Cæsarean operation. The success which attended the performance of the operation by Dr. Drew, who removed the tumor by the perinæum, has already been referred to. A similar case is recorded by Burns: both mothers were saved, although but one child was born alive. The tumors which formed the causes of obstruction in the cases of Siebold and Osiander, before noticed, might no doubt have been removed, without much difficulty to the operator, or danger to the mother. The cases that seem to be adapted to this means of relief are, doubtless, steatomatous growths; for they are more defined, less closely attached, and more moveable than other tumors. Scirrhous tumors, when they exist, or the scirrhous enlargement of the glands, are not fitted for the performance of this operation; while hydatid, encysted, and suppurative tumors may be diminished by the operation of puncture or incision. The operation of cephalotomy does not seem successful in saving the lives of the mothers in those cases in which it has been resorted to: in fact, if possible, I would rather remove the obstructing cause, than expose the woman to that violence which is necessary to deliver the child, even though the head be lessened as completely as possible: and rather than resort to the employment of such violence in effecting the delivery, I would prefer the Cæsarean section; as I think, if that were performed early, the mother would have a better chance of ultimate recovery."

F. *Pelvic Herniæ.*

The pelvic herniæ which may obstruct parturition are—vaginal herniæ—and perineal herniæ.

*Vaginal Herniæ.*—"The displaced parts either protrude between the bladder and the anterior part of the uterus and vagina (although its occurrence is not very frequent either in pregnancy or parturition), or below the posterior part of the canal and the rectum. When these displacements take place, they occasionally become an impediment to the progress of labour. The size of the protrusions differs. Smellie has related a case in which the finger only could be inserted between the hernial tumor and the symphysis pubis. This displacement does not often occur in primiparæ, although I have seen it in a first confinement: it more often takes place in females in whom there exists great laxity of the canal, from having given birth to many children, or from having undergone difficult labours. Chelius regards a posterior inclination of the pelvis as a predisposing cause. The exciting causes are, lifting heavy weights, violent strainings in evacuating the fæces or in child-birth, &c. The diagnosis of vaginal herniæ is highly important; the tumor is found to have formed suddenly, or it may gradually enlarge: it is of an ovoid or round shape, elastic, soft; and when pressure is made, it communicates a gurgling noise; if it contain intestine, it is larger in the standing than sitting posture; altogether disappearing when lying down; becoming painful to the touch when it has long been pressed upon by the child's head; and accompanied with nausea, vomiting, colicky, and other pains attending incarcerated herniæ. When the patient lies down, the tumor, as I have said, in most cases, returns spontaneously: in other instances it may remain; and its removal from the sac is accompanied with the gurgling noise which we know attends the reduction of other herniæ. If the patient cough, the hernial tumor will return, and may be again replaced. If the tumor contain merely omentum, it will give an unequal doughy feeling to the fingers: its shape is mostly cylindrical, its base wide, its formation more slow, and the patient feels a dragging from the scrobiculus cordis. The mouth of the womb is seated above and anterior to the tumor. It is sometimes difficult to distinguish vaginal hernia, especially when it is situated between the vagina and the rectum, from other tumors which form in that locality; but the history of the symptoms, the recurrence of the swelling, the change which takes place in the alteration of posture, the symptoms which accompany the delivery, and a careful examination, are the grounds upon which we should frame our diagnosis. In forming our prognosis where labour is thus complicated, we must be guided by the size of the protrusion, and the nature of the parts therein contained. In all cases, the child's life must be more or less endangered, from the narrowing of the passage, and from the protraction of the labour. The mother's life may be secondarily endangered (although she be delivered) from the sequences of inflammation of the intestines."

*Treatment.*—The hernia should, if possible, be returned, before the child's head occupies the pelvis. This may be attempted with the patient on her left side, or back, or even on her knees and elbows. When the hernia has been replaced, it must be kept so, till the head of the child occupies the pelvis. If the hernia should be strangulated, with the head pressing on it, we should at once deliver by the forceps, or open the head of the child, and finish delivery as fast as possible. The hernia must afterwards be treated on common surgical principles. The patient should wear an oval pessary, or one of caoutchouc.

*Perinæal Herniæ.*—The intestine descends to the perineum, between the rectum and vagina. It may be detected through both. In the taxis, the pressure may be applied through the rectum or vagina.

This concludes Dr. Lever's memoir, of which, with the exception of the cases, we have given an ample account. It is well worth the attention of every practitioner engaged in operative midwifery.

### III. An Inquiry into Certain of the Causes of Death after Injuries and Surgical Operations in London Hospitals. With a view to their Prevention. By Norman Chevers, M.D.

Such an inquiry is certain to be interesting, and may be useful. Notwithstanding the valuable facts and observations which have, from time to time, been communicated to the public, a complete investigation and exposition of the subject are desiderata.

Dr. Chevers remarks that, though great improvements have been made in the mode of performing operations, and in the regimen and treatment of patients, both before and after them, their fatality is still great. His remarks on the statistics of operations are just. Admitting their utility, he observes :—

"But, in one main point, these statistical observations fail in practical utility—in not admitting of being generally applied; for, as the results of operations are evidently greatly modified by locality and individual idiosyncrasy, they can scarcely ever be expected to correspond in any two situations or classes of persons. To illustrate the uncertainty which may attend the results of surgical operations, even when performed by one set of surgeons in different hospitals of the same town, I may quote a fact lately brought forward by M. Malgaigne, who states, that strikingly various degrees of success attend amputations in the several large Parisian hospitals; that in the most fortunate hospital for pathological amputations,* one death occurred in five; in the least fortunate, nine in ten; in the most fortunate for traumatic amputations, three deaths occurred in ten; in the least fortunate, all the patients who had been operated upon, died. It is evident that these results must depend upon some peculiar circumstances, either in the localities of the respective hospitals, or in the constitutions of the patients admitted to each; as it is stated that equal degrees of ill-success do not attend the same surgeons in their operations at different hospitals."

He proceeds to examine the causes of death after operations. They appear to resolve themselves into three distinct classes—the Primary, Secondary, and Remote.

"The first of these, or *Primary*, will include all fatal accidents which may occur during, or very shortly after, operations or injuries: such as, death from

Collapse,
Hæmorrhage,
The admission of air into the veins, or
The sudden occurrence of any internal lesion.

The *Secondary* include those fatal causes which are liable to come into action

---

* "Under the head of 'Pathological Amputations,' M. Malgaigne includes all those which are resorted to for the removal of ordinary disease: under the 'Traumatic,' those which are rendered necessary by wounds.—*Archives Generales*."

within a few hours or days after the receipt of injuries or the performance of an operation. They may be sub-divided into two sets :

"1st, Those which appear to depend upon some obscure lesion of the nervous system ; as, for example,

Tetanus,
Delirium tremens,
Irritative Fever.*

"2dly, Those which are attended with some manifest local change; such as,

Arachnitis,
Pleurisy,
Pneumonia,
Pericarditis,
Endocarditis.
Aortitis,
Peritonitis,
Purulent Arthritis,
Suppuration in the Liver, or other Abdominal Viscera,
Phlebitis and Arteritis,
Laryngitis and Diptheritis,
Enteritis,
Secondary Hæmorrhage,
Sloughing,
Erysipelas.

"Lastly, the *Remote* causes will comprise those which produce death after an interval of some weeks or months has elapsed, from the date of the operation or injury. The principal of these are,

Profuse Suppuration,
Secondary Fever,
Caries of Injured Bone, with Suppuration,
Phthisis.

"There are also several fatal causes which may come into action at almost any period between the time of the injury and the complete healing of the wound; such as, hospital gangrene, and various forms of endemic and epidemic fever."

Dr. Chevers informs us that his observations will apply principally to those causes of death included under the *Secondary* class—affections, mostly inflammatory or suppurative of surfaces or parts, unconnected directly with the original seat of disease or injury.

He quotes some authorities on the existence and prevalence of these secondary affections, hardly, however, doing justice to some, and rather understating, we conceive, what is known of them. He then proceeds to inquire into their ultimate causes, on which he concludes, and we quite agree with him, that the present state of our information is exceedingly unsatisfactory. After noticing the more prominent of the theories upon the subject, Dr. Chevers proposes, what, perhaps, he may be unwilling to admit to be his own. He says :—

"It has always appeared to me that there is probably one set of definite constitutional causes, which is ever ready to call these local mischiefs into fatal action, immediately upon the individual being subjected to any kind of unusual vicissi- or injury.

"There is a very large class of individuals in this metropolis, and probably in

---

* "I have here submitted to the custom which places Irritative Fever among the obscure affections of the nervous system ; but it is certain that cases in which death is attributed to constitutional irritation occasionally occur where the whole train of symptoms characteristic of this state appear to depend upon a condition of general Arachnitis, which is found upon dissection."

most of our densely-populated towns, in whom excessive labour, constant exposure to extreme changes of temperature, intemperance, and not unfrequently the action of syphilis and the abuse of mercury, have produced such a generally-diseased condition of the system, that its powers of reparation after injury have become almost entirely destroyed. It will indeed often happen that these individuals are for a long time enabled to follow their ordinary avocations without appearing to suffer much inconvenience; but so soon as they become the subjects of any injury, as a contusion, a fracture, or even a slight puncture or laceration, any surgical operation, sudden loss of blood, or, in short, any depressing or exciting influence which tends to produce increased derangement of their organs of circulation and elimination, they almost inevitably become affected with acute inflammation of the most deadly kind, in some one or more of the great cavities of the body; scarcely one of the serous or mucous surfaces (as I have before stated) being exempt from a chance of becoming affected. This state of the system, which is often spoken of under the vague and unmeaning term of 'cachexia,' is usually clearly traceable to a morbid condition of some of the principal organs of nutrition and elimination: the kidneys, liver, or spleen, have long been either in a state of confirmed disease, amounting to disorganization of portions of their structures, or have for a lengthened period remained in a condition of such intense functional derangement, as to be continually liable to fail in their actions upon the induction of any state of unusual constitutional excitement. Large numbers of patients of this description come under the surgeon's care: and I think I shall be able to shew that it is mainly owing to such a condition of the principal viscera as I have described, and not to the severity of the mechanical injury or to any fault in the mode of operating, that the greater number of deaths from internal inflammation, after accidents and surgical operations, generally occur.*"

To fortify his observations, he has referred to the Registers of the Museum of Guy's Hospital during the last fifteen years. To these results, of course important, he appeals. Our next extract will be rather long.

"One hundred and fifty-three cases of the kind were obtained from that source. Many of the subjects of these reports had undergone severe operations, or suffered from extensive accidental injuries; others had been the subjects of wounds or contusions of an apparently very trivial kind: still, the internal inflammations which destroyed life in most of the latter cases were generally as severe as those which proved fatal in the former instances, and frequently more so. In these 153 cases, death took place from—

---

* "It is of course undeniable, that, in certain situations, individuals who have previously enjoyed vigorous health die in great numbers in consequence of operations; but, in such instances, the fatal results are usually attributable to some unfavourable circumstances in the locality, or to the prevailing state of the atmosphere; and the fatal symptoms take on a nearly uniform character in every case. Thus, in some instances, tetanus has swept off large numbers of the wounded after general actions; in others, a form of low irritative fever has thinned the wards of military hospitals; and more than once, during the late war, it was found that, in certain ships, all those who were the subjects of wounds of any description became liable to hospital gangrene. But in the cases under consideration, the causes of death, although undoubtedly subject to be accelerated by bad air, confinement, &c., are evidently not mainly dependent upon such influences; as they may always be traced to a particular state of each individual's constitution which must have existed previously to his becoming the subject of accident or operation."

| | |
|---|---|
| Inflammation of secreting surfaces or internal organs (excluding the kidneys, liver, and spleen) in . . . . . . . | 134 |
| In the remaining 19, the patients died from other causes; such as tetanus, sloughing, hæmorrhage, suppuration, gangrene, erysipelas, diarrhœa, and the total deficiency of reparative action in the wound: and in one of these cases the precise cause of death could not be discovered . . . . . . . . | 19 |
| | 153 |

"In but a small proportion of the above 134 cases (in which the injuries or operations were followed by the occurrence of fatal internal lesions) were the inflammatory affections found to be confined to a single organ or secreting surface; but it was generally noticed that several important parts, and these often at a considerable distance from each other and from the seat of the primary injury, had become equally involved.*

"The following is a list of the various recent inflammatory lesions which were found to have occurred in the above 134 cases† :—

| | |
|---|---|
| Acute disease of the substance of the lungs, appearing in the form of inflammatory œdema, red or grey hepatization, abscess, or gangrene, was noticed in . . . . . | 47 cases |
| Bronchitis alone . . . . . . | 2 " |
| Pleuritis . . . . . . . | 35 " |
| Laryngitis and diptheritis . . . . . | 2 " |
| Meningitis . . . . . . . | 27 " |
| Inflammation, softening or abscess of the brain . . . | 9 " |
| Pericarditis . . . . . . . | 14 " |
| Peritonitis . . . . . . . | 52 " |
| Arteritis and aortitis . . . . . . | 4 " |
| Phlebitis . . . . . . . | 3 " |
| Inflammation of various portions of the intestinal canal (excluding cases of hernia) . . . . . . | 9 " |

* "It may here be inquired, were not the inflammatory attacks, in some of these cases, the necessary results of the injuries which the patients had received? In a certain proportion, this may have been the case. In about thirteen of the above instances, the nature of the injuries was such, that it was evident the patients could have no fair chance of recovery: in the whole of the others it appeared that there was nothing to render the patients' restoration impossible, had not severe inflammation or some other unfavourable change intervened. It is not usually to be supposed, in cases of simple fracture of the scull, fracture of the ribs, and operations for hernia, that arachnitis, general pleurisy, and peritonitis, will necessarily follow: these are results which must commonly be referred to some error in the patient's constitution. Again, in given cases of laceration of the brain, and wound of an intestine, the injuries may in themselves be necessarily mortal; but where, after the patients' deaths, pneumonia is found to have been set up in the first case, and pleurisy in the second, we have just grounds for inquiring whether some previous fault in the constitution has not caused these lesions to be superadded to those which would naturally result as the immediate local effects of the injuries."

† "It will be observed, that the figures in this Table merely denote the number of times particular inflammations were found to have occurred, and have no reference to the total number of cases. Thus phlebitis is stated to have appeared in three cases, and arteritis in four; but there were, altogether, only six cases of vascular disease; in three of which there was arteritis, in two phlebitis, and in one arteritis and phlebitis combined."

| | | |
|---|---|---|
| Suppuration in the substance of the Psoæ muscles . . | 2 | cases |
| Acute purulent synovitis . . . . . . | 1 | „ |
| Inflammation of the tunica vaginalis . . . . | 1 | „ |
| Cystitis . . . . . . . . . | 8 | „ |

With regard to the state of the kidneys, liver, and spleen, I found that

| | | |
|---|---|---|
| The kidneys were observed to be in a state of marked disease, either presenting remarkable congestion, softening, mottling, or the granular or cystiform alterations in . . . . | 72 | cases |
| The appearances of the kidneys were not mentioned (usually from the autopsy having been only partial) in . . . | 44 | „ |
| These organs were stated to be without any apparent disease in . | 26 | „ |
| The condition of the kidneys was doubtful in . . . | 11 | „ |
| | 153 | „ |

"Of the above cases, in which the kidneys were either not examined, found healthy, or considered in a doubtful state, there was marked disease of the liver or spleen, or of both these organs, in 21 cases—giving a total of 93 cases, in which one or more of these important organs was found in a state of lesion.*

"It was observed, that of the 134 cases in which the patients died of internal inflammations, there was also superadded marked disease of the kidneys, liver, or spleen, or of all these organs combined, in 90.†

"In a rather large proportion of these cases, the disease of the liver, spleen and kidneys had evidently existed for a very considerable time previous to the patients receiving the wounds or injuries which became the apparent primary causes of death: but in very many (and this was especially observable in the renal cases) the changes were evidently of so recent a nature, as to render it probable that almost immediately after the operations or accidents, either visceral disease had been excited from a latent to an active condition, or that a state of acute congestion had suddenly been established in organs which had hitherto been suffering merely from chronic degeneration."

One of Dr. C's principal objects in submitting these remarks to the profession has been, to direct attention to the frequency of renal disease in those who perish from the secondary effects of operations or injuries.

We will not go into our author's reasoning upon the matter. The fact, *if* a fact, finds much of its own explanation. The thing is, to test the fact's reality, and that can only be done by actual observations in the dead house. For our own parts, having paid some attention to the subject, we are inclined to believe that Dr. Chevers overrates the prevalence and importance of visceral disease as a cause of these secondary affections. We are, by no means, disposed to deny the influence which such disease would exert, nor its occasional existence. But, of its frequency to the extent contended for by Dr. Chevers, we repeat that we have our doubts.

For our own parts we believe that, independently of internal and constitutional causes, the nature of the local mischief exerts an important influence. If that is

---

* "From the character of the symptoms and the nature of the inflammatory lesions of which many of the patients died, I am convinced that renal disease would have been discovered in a considerable number of those cases in which the state of the kidneys was not observed, had those organs been examined after death."

† "While engaged in taking notes of the above cases, from the Post-mortem Registers, I met with the following observation by Dr. Hodgkin, appended to the case of a man who sunk after lithotomy, about fourteen years ago, and in whom mottling of the kidneys was discovered after death:—'This condition of the kidneys was also noticed in another patient who died after the operation of lithotomy; and in others who have sunk after operations and injuries.'"

of such a nature, that matter formed cannot get free exit, secondary inflammations and depositions are common—if it is capable of being evacuated freely, they are comparatively rare. We shall return to this subject more fully hereafter.

Dr. Chevers alludes to the tendency to secondary hæmorrhage after operations in patients who labour under renal disease. He also gives some further examples of the manner in which renal disease gives rise to fatal symptoms in surgical cases.

" Mottled kidney is a very frequent attendant upon old strictures of the urethra. Persons thus affected are liable to be seized with shivering, vomiting, a quick pulse, and an anxious expression of countenance, but often without much localized pain; and to die with peritonitis or inflammations of other serous membranes, cystitis, and often with suppuration below the peritoneum at the base of the bladder, sometimes following the course of the ureter, and occupying the cellular tissue surrounding the kidney.

" Such individuals are also apt to be suddenly attacked with inflammatory œdema of the lungs and cerebral effusions, ending in coma and death.

" As, in cases of this description, the above symptoms not unfrequently occur as the results of the simple introduction of a bougie or sound, it becomes of importance to ascertain, if possible, the state of the urine in every case of stricture, previously to having recourse to the use of instruments for its treatment.

" Adult patients suffering from stone in the bladder are often the subjects of an active form of Bright's disease. Where this is the case, the operation of lithotomy seldom fails to be rapidly followed by fatal inflammations of serous and other structures in various parts of the body; and, as I have already mentioned, there is in such cases considerable tendency to the occurrence of secondary hæmorrhage.*

" Syphilitic patients are liable to be unexpectedly attacked with œdema glottidis, purulent inflammation of the larynx and diptheritis, or with rapidly-destructive forms of pleurisy, pneumonia, and peritonitis. It is generally found that the individuals who are thus destroyed have long been of intemperate habits, have suffered from the abuse of mercury, and become the subjects of mottling or granular disease of the kidneys.

" Persons suffering from asthenic anthrax are apt to become affected with acute pleurisy and other internal inflammations; the effused fluids occasionally taking on a sanguinolent aspect. I have found that patients who have died in this manner have been the subjects of marked renal affections, similar to those noticed above.

" Other patients, while under treatment for various kinds of surgical diseases, not unfrequently die from the effects of acute inflammatory attacks, ascribable to degeneration of the kidneys and of the other solid abdominal viscera.

" From facts already dwelt upon, with regard to the effects of wounds upon patients suffering from the different stages of Bright's disease, it becomes a point of extreme importance to be borne in mind, in a medico-legal point of view, that an injury, in itself of a most trivial kind, is liable to be followed by fatal results, should the subject of it be suffering at the time from either acute congestion, mottling, or granular degeneration of the kidneys."

Most of the preceding statements are, we believe, not unfamiliar to experienced surgeons, and the morbific influence of renal disease is both extensive and indisputable. Whether it operates quite so universally as Dr. Chevers supposes may probably not be so certain.

Dr. Chevers urges what cannot but be considered a judicious recommendation—that every patient, on entering the surgical wards of a hospital, should have

* " Mr. Key, I am informed, observes, in his Lectures, that he has scarcely ever seen a fatal case of lithotomy in which there was not discovered organic disease of some of the abdominal viscera, and more especially of the kidneys."

the condition of his urine tested. To establish the practical value of the advice, he urges the following sound considerations :—

"It is to be feared that the more confirmed forms of renal disease never undergo a complete and permanent cure; but it cannot be doubted, that during the earlier stages of the affection, while the glands are still merely in a state of great vascular turgescence (a period, by the bye, at which I believe inflammations of the serous membranes to be especially liable to occur), the disease is certainly amenable to remedies; and although, where there is reason to suppose that the kidneys have undergone a permanent structural change, every injury is attended with considerable risk to the patient, it is well proved that, even then, the disease of these organs may be rendered passive and kept down, the urine being, for a time at least, restored to its natural character. Under such circumstances, operations may not be productive of such certain danger. I have seen cases of patients suffering from stone at Guy's Hospital, where, some weeks previous to the operation, the urine had been albuminous; but this condition having yielded to remedies, lithotomy was performed, and the patients recovered without any marked bad symptoms. There can scarcely be a doubt that in these cases an operation undertaken while the kidneys were in an irritable state would have been unavoidably fatal."

He would, in all cases, then, test the urine, and examine the condition of the liver and spleen. It sometimes happens that decided renal disease is not attended with albumen in the urine. But the surgeon, if he has tested that, has done his best to arrive at the truth, and no blame can attach to him. Dr. Chevers adds :—

"Even where the urine has been found healthy immediately previous to the operation, I believe that there is still great necessity for testing its condition, at intervals of a few hours, during a considerable time after the patient has been removed from the operating-table to his ward; for the extremely recent appearance of the morbid changes observable in the kidneys of many of those who have sunk from internal inflammations after injuries has (as I have already stated) led me to believe that these organs often remain with merely a tendency to derangement until the disturbance consequent upon the operation causes them suddenly to take on a state of active disease."

### IV. Observations on the Structure, Functions, and Diseases, of the Coronary Arteries of the Heart. By Norman Chevers, M.D.

After a description of some peculiarities in the structure, and some observations on the functions of the coronary arteries, for which we must refer to the original, Dr. Chevers offers an account of their diseases.

They are such as affect the aorta, from which they generally extend in a greater or a less degree. But the coronary arteries seem to have also some morbid changes more peculiar to themselves.

They may partake in nearly all the changes resulting from acute aortitis, and have been found obstructed by recent concretions of plastic lymph. But they also present a frequent redness from staining and transudation.

The orifices of the coronary arteries are peculiarly liable to cartilaginous or ossific deposits. It is not rare to find deposits of this nature partially surrounding and obstructing these openings, and occasionally encircling them with a tumid ring, which nearly, and in some cases entirely, precludes the entrance of blood.

"But the mouths of both these vessels are not usually found equally narrowed; and where only one of them is affected, it is not certain that any very serious consequences are produced; as the communication by anastomosis between the right and left appears to be sufficiently free to enable the sound vessel to supply all the branches below the impediment in the diseased one; and closure of one vessel has been noticed to be followed by dilatation of the other. In the adult, their interiors are seldom entirely free from minute opacities, which are usually arranged in striæ corresponding to the course of the longitudinal fibres of the subserous laminæ between which they are situated. In advanced life, it is common to find bony deposits scattered at intervals throughout their whole extent: some of these frequently attain a very large size, appearing as narrow elongated masses, lying parallel to the axes of the vessels; rarely surrounding the canals with complete calcareous rings, as do the old deposits of the smaller arteries of the extremities, but often attaining a bulk sufficient either to close the vessels completely or to leave a very narrow and irregular passage for the blood; while the less rigid portions of the tubes become greatly dilated, and in this state occasionally suffer rupture. Except in extreme cases, this condition of the coronaries is not invariably followed by much atrophy of the muscular substance of the heart; for as the minuter ramifications of the arteries generally continue comparatively free from disease, a sufficient quantity of blood permeates the strictured vessels (providing the circulation remain tranquil) to maintain the nourishment of the organ. The coincidence of this state of the coronary arteries with the symptoms of angina has long been observed; and it appears probable that the sudden attacks of syncope, to which aged persons are frequently liable, are, in a great proportion of cases, attributable to the same cause."

Large atheromatous collections between the layers of the sub-serous tissue are not frequent But they may occasionally be pressed through ulcerated openings in the internal membrane of the vessels, and have been mistaken for pus.

The coronary arteries are liable to permanent dilatation, either throughout their whole extent, or in varicose bulgings, like those of the superficial arteries of the scalp.

"Their tortuosity is also greatly increased, and occasionally (the surrounding adeps becoming absorbed) they project so greatly from the surface of the heart as to appear to be attached to that organ only by a fold of pericardium. Occasionally, this dilatation is followed by rupture of one of the arteries; of which fatal result examples may be met with in almost every collection of morbid anatomy: but the occurence of that accident generally appears to be long delayed by the increased strength which the sub-serous laminæ acquire during the time in which the vessel is undergoing the process of dilatation; and it is probable that, in most of the cases where this accident takes place, it is immediately dependent either upon inflammatory softening or ulceration of the inner tunics of the vessel."

Dr. Chevers goes on to observe:

"I believe one of the most frequent causes of dilatation of coronary arteries to be, the deposition of a large quantity of fat within the interstices of the muscular tissue of the heart, which appears to produce here (as a similar process clearly does in other parts of the body) a diminution in the capacity of all the minuter capillaries distributed within the organ; and hence the main trunks, although sufficiently furnished with blood, become incapable either of transmitting their contents with freedom, or of supplying the heart with its proper nutriment; and accordingly, the large branches of these arteries suffer marked dilatation, while the cardiac muscular tissue becomes pale, softened, and atrophied: and hence arises one of the most frequent causes of rupture of the heart. It has become a subject of remark, that in the cases where death occurs from sudden rupture of one of the heart's cavities, the whole of the surface and furrows of the organ will generally be found loaded with adipose tissue; and (judging from a considerable number of specimens in the various metropolitan museums) we may also add, with the coronary arteries considerably dilated. It may not be altogether unimportant to bear the above facts in mind, with regard to the application of remedial measures in these cases. It is by no means unusual to find elderly persons of obese habits of body complaining of violent palpitation, with sensations of impending suffocation, after any sudden exertion or emotion, the application of cold to the surface of the body, or, in fact, any action which tends to determine an unusual supply of blood to the heart. In these persons, the pulse is usually weak, while percussion and auscultation shew that their hearts, although large, act feebly; the sounds being indistinct, but free from irregularity or other abnormal character. This train of symptoms is probably often dependent upon an advanced degree of that condition of the heart which I have last described; and I have little doubt that the difficulty of breathing, which nearly all extremely corpulent persons experience upon unwonted exertion, is mainly attributable to less degrees of the same changes. Acting upon this course of reasoning, I have found that the adoption of a plan of treatment calculated at once to procure absorption of a portion of the superfluous fat of these patients, and to diminish the quantity of their circulating fluids, has been followed by an acquisition of increased cardiac power, as evidenced by a stronger pulse and an entire cessation of the suffocative attacks during very long intervals."

The latter recommendations deserve attention, and correspond with what we have ourselves observed.

### V. A Case of Glanders in the Human Subject. By H. M. Hughes, M.D.

Dr. Hughes observes that patients are not unfrequently admitted into the hospital who are supposed to have been infected or inoculated by poison, derived from the lower animals. The symptoms and the progress of the case are too various to admit of a faithful general history being given of them. The following sketch appears to us to be graphic and true.

"One feature is, however, common to them all. The patients have been all more or less directly or indirectly, exposed to the liability of inoculation by putrid or diseased animal matter. One of them, a short time before his illness, was engaged in moving some barrels of salt meat, the odour of which proved that the antiseptic processes adopted had not been effective in preventing partial putrefaction; others have been occupied as tanners, knackers, butchers, horse-keepers, cab-drivers, or coachmen; or have been employed in packing or handling imported skins, or imperfectly cured animal provisions.

"These patients have fever of considerable but very variable intensity, often remittent, frequently irregularly intermittent, and constantly accompanied with a moist state of the skin. They almost always complain of severe pains in the back, limbs, and joints; and often believe and represent themselves to be suffering from rheumatism. The complaint has not, however, the general aspect of that affection. The joints are not, in the first instance, swollen, red, and tender; the tongue has not the loaded, white, moist, and flannel-like appearance; and the skin, though moist, is not covered with a copious perspiration of the peculiarly sour odour common in that complaint. Pain of the head, and especially of the forehead, is often troublesome; delirium exists at night; the tongue is somewhat dry and red, at least at the tip and edges; the conjunctiva is injected; and the whole of the mucous membranes congested and irritable—symptoms which lead to the probable supposition that the disease is simple continued fever. But the absence of some of its ordinary accompaniments causes doubts to arise in the mind of the attendant: the skin is not constantly dry, pungent and hot but is at one time hot, and at another moist, greasy, and of natural temperature; there are no maculæ, and no petechiæ: and though the face is flushed, the countenance has not the heavy, stupid, half-drunken, 'puzzled' expression of common fever. While the physician is perhaps still undecided as to the origin and nature of the malady, a local swelling occurs upon the trunk, or more commonly about one or more of the joints; or erysipelas appears upon the forehead, cheek, or neck. The tumor slowly suppurates, or the erysipelas is followed by an ill-defined abscess of the cellular membrane. The parts heal, and the patient for a time appears to improve in health and strength; when tenderness is felt in another part, and one or more tumors are again discovered, slowly suppurate, and discharge their unhealthy contents. The parts heal, and the patient is again temporarily restored: his improvement is but brief; his quiet is but of short duration; other tumors again and again appear: the process is repeated, till his spirit is broken, his energy lost and his constitutional power destroyed. The tumors have usually no definite arrangement, and certainly are not confined to the course of the veins and absorbents: they are, on the contrary, seen at the same time, or in succession, upon the right arm and left leg, the axilla of one side and the groin of the other, the instep, and the sub-maxillary space. Most commonly the poor patient, already nearly exhausted by the repeated drains upon his system, caused by the discharge from the abscesses, is ultimately carried off by an attack of diarrhœa. More rarely, an individual, after a long and tedious convalescence, slowly acquires strength, and is at length discharged; cured indeed of his complaint, but in a condition so shattered as to be unable for some time to resume his ordinary occupation."

These cases, as Dr. Hughes observes, bear a strong resemblance to those which have been reported as instances of chronic farcy; but their common origin has not been proved.

Only two cases of well-marked acute glanders have been admitted into Hospital. One occurred in Dr. Hughes's absence, and some particulars of it were communicated to the Medical Gazette. The second case is now narrated.

*Case.*—" Michael Ginivan, aged 40, a horse-killer, was flaying the leg of a horse recently killed, when his knife slipped while his attention was temporarily withdrawn, and inflicted a wound in the fore-arm of the *left* side. On the next day, a cord-like enlargement made its appearance, leading from near the wound to a painful swelling on the inner border of the biceps muscle, just above the elbow." He went to a surgeon, who dressed the wound and gave him some aperient. He stayed at home and thought little of the matter for two or three weeks, " when he began to suffer pains in different parts of his body resembling those of rheumatism, and painful swellings arose in his *right* arm and leg.—Mr. Comley seeing him in the yard, to which he had gone to see his master, and struck by his peculiar appearance, gave him a Letter for the Eastern Dispensary; and took him under his own care, September 23, 1842, the twenty-fourth day after the accident.—He was robust, muscular, and apparently of good constitution; and for the last ten months has adopted 'the total-abstinence principles,' though he had previously been in the habit of drinking to excess. The countenance was anxious and depressed, but 'irritable, as if labouring under the effects of poison;' the eyes sunken; conjunctiva slightly jaundiced; pupils dull and sluggish; intellect clear; tongue flabby; bowels regular, pulse 60, with little power: he had no appetite: the respiration was not disturbed. The wound in the fore-arm was superficial, with an inflamed surface, and hardened base: two or three hardened lymphatic vessels led from the wound to a large and pointed tumor on the inner edge of the biceps, above the internal condyle. There existed also a deep and fistulous wound over the first phalanx of the little finger. The glands in the axilla were not affected. A tumor similar to that on the *left* arm existed on the centre of the *right* fore-arm, and also on the *right* thigh: they were all acutely tender to the touch.—Ordered, a nutricious diet, with a pint of porter and six ounces of wine daily; to take an alterative powder of rhubarb and calomel every other night, and an aperient draught the following morning; decoction of bark ℥iss. and carbonate of ammonia gr. v. three times a-day."

25th day. Pains in the whole body—unable to walk—pulse 80, soft.

After this he slowly improved till the 35th day, when he was not so well. "The pain of the right leg was very severe, and prevented both motion and sleep; he had also a pain in the back, which was referred to the right kidney: the urine was not examined: he had some cough, unattended with expectoration, but accompanied with some crepitating rattles in the posterior and inferior portion of the *right* side of the chest: the countenance was again anxious; the pulse 80, full and sharp; the skin moist; the bowels regular.—Ordered to discontinue the wine; to repeat his alterative and aperient medicine; and to take sesquicarbonate of soda gr. x. tinct. of orange ʒj., and compound infusion of gentian ℥j. three times a day."

37th day. Cough has subsided—looks better. To resume wine.

41st day. Not so well. Acute pain on the inner side of the *left* knee. Eight leeches, and poppy fomentations. P.

42nd day. Small tumors on the left leg—those of arms more swollen and painful.

44th day. Pains of entire body—tongue red in centre, brown-furred at edges—countenance anxious. To take a pill of hydrochlorate of morphia gr. ss. and blue pill gr. ij. every night, and decoction of bark ℥j. with compound spirits of ammonia ʒss. three times a-day.

46th day. Symptoms typhoid—leg and knee much swollen and acutely painful.

47th day. Admitted into Guy's Hospital. "There existed a considerable swelling about the *right* knee, which was rather red, painful, and tender under pressure, but appeared to contain no defined collection of fluid. Upon different parts of his arms and legs he had six or eight tumors, oval, soft, and about the size of half a pigeon's egg; some of which only were tender, but all evidently fluctuated under pressure, and over all the skin itself was of the natural colour and appearance; the tongue was clean and moist: the pulse 84, of moderate power; the respiration unaffected. Ordered, sesquicarbonate of ammonia gr. v. decoction of bark ℥iss. every six hours.—A draught, with tinct. of opium, ♏xx. in camphor mixture, at bed-time.—Two pints of porter and ℥iv. of wine, daily; with good beef-tea and arrow-root for food; and fomentations to the knee."

48th day. Some wandering delirium in the night. Knee less swollen and tender, slight increase in number of small subcutaneous tumors.

49th day. Pulse increased in frequency, diminished in power. "The *left* eye was closed by a purplish-red tumefaction of the eyelids: the tongue was rather dry, and at the tip and edges more red than natural: the bowels had been opened twice: the knee was much less swollen; and the tenderness about it was now trifling; but the small tumors had increased considerably in number. Several large flat elevations of the cuticle, containing an opaque yellow fluid intermixed with some which were smaller and acuminated, now existed, thinly scattered over the extremities."

"Sesquicarbonate of ammonia gr. v. Compound tincture of bark ʒj. Wine of opium ♏v. Infusion of serpentary ℥iss. every six hours.—The night-draught to be repeated, with tincture of opium ♏xxx. To continue the porter, and to have ℥iv. of brandy instead of wine."

50th day. Somnolency. "The *left* eyelids were more swollen, and the left side of the face appeared œdematous. The *right* eyelids were also swollen, though to a less extent. The *right* knee had assumed its natural size and appearance; but the pustules had now appeared upon the face and trunk, and their number had very much increased upon the extremities. When more advanced, they were rounded, plump, yellow, and about the size of half a large split-pea: those which had recently appeared were smaller and rather pointed, containing yellowish opaque fluid, like some forms of varicella. Four only existed on the face; one of which was on the forehead, one on the left eyelid, one on the top of the nose, and one on the chin. When the right eyelid was raised, there exuded a little clear colourless fluid. From the nose spontaneously flowed a scanty, reddish-brown, and rather viscid secretion, which appeared to obstruct the respiration. No enlargement of the absorbent glands of the axilla, groin, under the jaw, or of other parts, could be discovered. The skin was now perspiring very freely, and the pulse was very feeble and frequent. The state of the pupils could not be ascertained, in consequence of the swelling of the eyelids.

His external appearance now much resembled that of the patient of whom the model is preserved in the Museum, though the characters of the complaint were generally less strongly marked. Ordered to repeat the mixture every four hours without the opium: to have ℥viij. of brandy: and to continue his porter, beef-tea, &c.

"He continued in exactly the same state, excepting that he was very thirsty and drank freely till 9 o'clock P.M., when he had a little rattling in his throat, suddenly became pale, and ceased to breathe. It appeared probable that he was sensible to the last; as, though he could not be understood, he tried to talk, when his wife spoke to him in Irish. Permission was not granted to inspect the body, which was speedily removed by the friends."

It may be observed, in reference to the cause of these symptoms, that a horse affected with "black farcy" had been admitted into the yard the day before the accident, and glandered horses were frequently slaughtered at the establishment.

Dr. Hughes concludes:—

"The history of this person's disease before his admission into the hospital bears a striking resemblance (excepting the formation of abscesses) to those referred to at the commencement of this paper. On the day of his death, his external appearance was very similar to that of the person formerly modelled in the Museum. It is to be remarked, that the small tumors of the skin appeared in numbers only during the last few days of the disorder, and that the pustules arose only three days, at most, before that event: it is also remarkable that at the same time the swelling near the knee, which obviously contained fluid, subsided. The treatment was adopted with the view of keeping up the power of the patient while the poison was thrown off from the system, or 'the disease wore itself out;' but when the complaint assumed an acute form, it had little, if any, effect. Hitherto all the cases of the acute disease have, I believe, proved fatal. Is it not time, and would it not be expedient and proper, to adopt other means of cure; such as the free administration of mercury, and giving a free and early exit to all accumulations of matter?"

### VI. OBSERVATIONS ON THE BLOOD, WITH REFERENCE TO ITS PECULIAR CONDITION IN THE MORBUS BRIGHTII. By GEORGE OWEN REES, M.D. F.R.S. Physician to the Pentonville New Model Prison.

It appears that Dr. Rees had many opportunities, during the last Summer, for investigating the condition of the blood in the *Morbus Brightii*. Of those opportunities he has availed himself.

Prior to entering on the morbid condition of that fluid, he thinks it necessary for comparison's sake, to premise the result of a few experiments on the anatomy and mechanical relations of the blood-corpuscle.

The following remark is not very flattering to the microscope or microscopists —worse than that, it is a true one.

"It is hoped that the experiments about to be described may shew the necessity for a correct knowledge of physical structure on the part of those who are occupied in the chemical examination of the blood; and, also, that it may appear how we occasionally possess means of proving on large masses the views to which we have been led by microscopic examination—a method of inquiry which, valuable as it certainly is, must always be received with the distrust naturally felt towards a means of investigation so tempting to the imagination, and which, it is to be feared, has already been productive of much mischief in the hands of the ingenious and unscrupulous."

Our present limits scarcely justify us in entering on the microscopic examination of the blood in health. We must, therefore, pass to the state of that fluid in renal disease.

The following appear to be the principal features to which attention should be drawn:—1. The excessive quantity of water in the blood. 2. The existence in the blood of one of the ingredients of the urine. 3. The existence of the same ingredient of the urine in the milk, and also in the fluids effused into various serous cavities. 4. The absence or deficiency in the urine of one or more of the natural ingredients of the excretion. 5. The general watery condition of the urine. 6. The existence of albumen in the urine.

Dr. Rees observes:—

"When considering the part taken by the blood in producing the Morbus Brightii, it must not be too rapidly concluded that those changes which are observed in more advanced stages of the disease are identical in kind, and differing only in degree from those occurring at the commencement of a severe and fatal case; or that they are the cause of the symptoms, terminating in perfect recovery, which we so often observe in mild cases of anasarca with coagulable urine following scarlet-fever. The diseased conditions of the blood noticed in the Table may, however, I think, well be considered the cause of the train of secondary symptoms attendant on the Morbus Brightii: and the first morbid condition induced may be (as has been rendered more than probable by the late ingenious researches of Mr. Robinson) simply a congested state of the kidney—a mechanical derangement of circulation—giving rise to a filtration of the albuminous matters of the blood into the urine; a drain on the system which, by impoverishing the vital fluid, may, in its turn, make the blood a cause of further symptoms, such as would never have developed themselves had not the primary disease been manifest. All, indeed, that we know of the history of this degeneration of the kidney, the mild character of some of the cases, and the facility with which the disease, as following scarlatina, admits of cure—tends to show, that in the commencement the blood may be perfectly healthy, and the albumen in the urine the result of congestion by blood in its normal state.

"There is, however, a fact in the history of this affection which does not render it altogether improbable that the presence of an excess of water in the blood may, in some cases at least, assist in bringing about the effusion of serum into the urine. I allude to the frequency of a dry skin, observed in some early cases. The probability that such a state of the cutaneous surface acts as a cause is considerably increased by the tendency to this kind of dropsy after the cutaneous surface has been involved by an attack of scarlatina, which produces a form of Morbus Brightii, for the most part easily admitting of cure. The difficulties which must necessarily occur in freeing the blood of water when the action of the skin is lessened or entirely stopped must be considered as very great, when we remember the large quantities of fluid daily given off from the cutaneous surface, and the small excess only in the quantity of urine characterizing any form of Morbus Brightii;—the greater number of cases, indeed, passing less than the natural quantity."

Yet, he does not absolutely contend that congestion alone is incapable of producing coagulable urine; though he thinks that the tendency to it will be increased by dilatation of the blood.

"The secondary symptoms of the Morbus Brightii—such as effusion into the large serous cavities or the ventricles of the brain, general cellular effusion, and the peculiar anæmic appearance which, even when no swelling of the face exists, is frequently so characteristic as to attract the practised eye—are easily explicable as results, when once we are acquainted with the watery state of the blood, and the physiological conditions necessary to preserve the integrity of the blood-corpuscle. The obvious mechanical assistance which an excess of water must afford for the production of general effusion needs no comment; but it may be a matter of difficulty to some, to explain how it is that the blood loses its red colouring matter; and which, to be clearly understood, requires an insight into the more minute changes occurring in the fluid, as a result of its aqueous condition. I have before shewn, that if the chyle does not accommodate itself in relative specific gravity to the blood, the necessary endosmodic actions cannot take place between the two fluids; and we may consequently expect difficulty in the production and growth of the red corpuscles, inasmuch as the iron cannot be supplied for the formation of colouring matter, the ferruginous serum of the chyle no longer entering through the membrane of the blood-corpuscle in virtue of its less specific gravity: and I think it may be maintained as the correct view, that this is really the cause of that great diminution in the proportion of colouring matter observed in the blood of patients affected with the advanced stage of the Morbus Brightii. This diminution in the proportion of red corpuscles does not occur in early cases of the disease, however confirmed in character: an example of which may be seen by referring to the history of George Moore, 14 Job Ward, a mild case, probably admitting of permanent cure, in which the blood contained more than the normal proportion of fibrin and corpuscles; the albumen being very deficient, the serum light, and the water of the blood in about its natural quantity. We here see the first effects of the disease—the blood becoming deprived of its albuminous ingredients; a condition which, if it continue, will produce the next change; viz. a deterioration in the specific gravity of the contents of the corpuscle, owing to the liquor sanguinis becoming lighter, and endosmosing that structure: this state again soon succeeded by a lessening of the number of red corpuscles, attributable to the requisite actions no longer taking place on the part of the chyle, in the manner described in a former part of this Paper. The occurrence of inflammation in this disease, as will be seen by the analysis of the blood in the case of Holywell, produces the usual increase in the quantity of fibrin in the blood: this, however, did not happen to any considerable degree in the case of Charles Scott."

The existence of urea in the blood and effusions, and in the milk of a patient, has been satisfactorily proved. To obtain urea from milk, the following appears to Dr. Rees the best process:—the milk must be evaporated to dryness; and then several times digested with æther, which will extract the whole of the fatty matters, together with the urea; the latter being easily separable by heating the dry ethereal extract with water, and stirring it well during the digestion. After this process the urea exists dissolved in the water, which may be poured off from under the fatty matter, the latter having caked above the liquor on cooling.

Dr. Rees thinks that, as regards the urine, the deficiency of urea, and occasional deficiency or absence of lithic acid, its watery condition, and the presence of albumen, the two former states may in all probability be attributed to the derangement of the kidney alone; and the two latter, in some measure, to the condition of the blood. He does not know that the total absence of lithic acid from the urine has been before observed.

"Albuminous urine, when viewed under the microscope, exhibits granules and corpuscles of varying form and size; the larger of which might be mistaken for the pus-globule by careless or inexperienced observers. The true source, however, of these bodies is, in all probability, the serum of the blood, which I find deposits analogous granules and corpuscles when diluted by a liquor of light specific gravity; which may easily be proved by pouring distilled water on serum, and submitting to the microscope the precipitate which collects after a few hours have elapsed. In other respects, coagulable urine presents the ordinary appearances under the microscope; the solid ingredients or crystallizable products exhibiting, when present, their usual characteristics. Some specimens shew very well the large form of granulated mucous globule known as the secretion of the prostate."

---

### VII. Account of Observations made under the superintendence of Dr. Bright, on Patients whose Urine was Albuminous: By George Hilaro Barlow, M.A. and M.D. With a Chemical Examination of the Blood and Secretions. By G. O. Rees, M.D.

Dr. Bright observes:—

“The few following pages will be found to contain the record of the first experiment which, as far as I know, has yet been made in this country to turn the ample resources of an hospital to the investigation of a particular disease, by bringing the patients labouring under it into one ward, properly arranged for observation. I fear that the attempt has been less effective than it might have been, and than, I trust, others will hereafter prove; but if this is the case, the imperfection must fairly be ascribed to my want, not so much of zeal as of time, for carrying out, in its fullest extent, a most interesting experiment.

“The Clinical Wards of Guy's Hospital seemed to afford a most appropriate opportunity for the object in view; and I applied to the Treasurer of Guy's for permission to occupy them after the Clinical Session had concluded, in May. Mr. Harrison entered heartily into the plan, and offered every facility. Accordingly, it was determined, that from May to October I should be allowed the full use of the Male and Female Clinical Wards; and all my colleagues readily granted permission to select any cases from the other wards which I might consider likely to promote the object.

“Dr. Barlow, with the greatest readiness, undertook to make up for my neglects; whilst Dr. Rees took charge of the chemical part of the inquiries: and it is entirely to these two that our Reports are indebted for the connected account of proceedings which the present communication contains.

“Our establishment then consisted of a female ward with eighteen beds; a male ward with twenty-four beds; a room between the two wards for the meeting of the physicians and pupils, and for the registry of the cases; and a small laboratory communicating with the middle room, fitted up and decorated entirely to our purpose.

“Dr. Barlow and myself were in charge of the medical treatment of the cases, with the assistance of Dr. J. T. Francis, Mr. J. H. Browne, and Mr. Allen Williams, as Clinical Clerks, whose duty it was to take daily reports.

“Dr. Rees had charge of the laboratory, in which he was assisted by Mr. Pearce; and, thus prepared, we began our operations, and proceeded pretty steadily with them.

“The objects which we proposed to ourselves were, to examine as far as it was possible, the changes which accompanied the secretion of albuminous urine in the various functions and secretions of the body; whilst at the same time we registered the various circumstances connected with the origin, progress, and treatment of the disease;—a disease than which there is certainly none which offers a more extended field for careful and well-directed observation.”

The Paper contains the particulars of 34 cases, an elaborate table, and several plates. It would be quite inconsistent with our limits or plan to go into such details on a complaint on which so much is daily said. The utmost we can do is to notice some facts or inferences that appear of interest.

After relating seven cases, Dr. Bright makes some remarks. He first touches on the cerebral complication of this disease.

“The two cases, in which the complication consisted of affections of the encephalon, illustrate forcibly the danger of the sudden invasion of cerebral symptoms; which always exist where albumen is present in the urine, in any quantity, however small, accompanied with a diminution in the specific gravity of that secretion; a danger which is not diminished by the absence or the subsidence of any dropsical swelling: for, in the former case, it is worthy of notice, that the quantity of albumen in the urine was but small throughout, and that the dropsical effusion had nearly subsided before the more alarming symptoms manifested themselves; and in the second case (that of Henry Stanley), the anasarca was much diminished, and the urine improved in every respect, except its specific gravity, just at the time that the fatal attack occurred. Indeed, I think that, from a careful observation of no very limited number of cases of this disease, I may pretty confidently affirm that in those cases of albuminous urine, in which there is little or no dropsical swelling, with but a moderate or even scanty deposit of albumen, the skin being at the same time moist and perspirable, but the urine defective in its solid contents—as evinced by its light specific gravity, and the absence of urinous odour upon the application of heat or nitric acid—there is more especial danger of the sudden invasion of disease in the brain or its membranes. In one or two very well-marked cases of this kind, almost the only dropsical effusion has been a sort of watery chemosis, produced, as I believe, by anasarca of the cellular membrane under the conjunctiva."

He then adverts to another and an interesting question—the relation of cardiac hypertrophy and renal disease to one another.

"The origin, however, of this hypertrophy is a matter of some difficulty, as well as of some importance, as the question has been raised: and it is certainly one deserving of grave consideration.—Whether, in cases of this kind, where disease of the heart co-exists with disease of the kidney (a coincidence which is observed so often as to render it in the highest degree probable that the two lesions bear some necessary connexion with each other) the former or the latter is to be regarded as the primary affection—whether, in fact, the renal disease is any thing more than the result of congestion produced by mechanical obstruction—or whether the disease of the heart can be in any way shewn to be the result of that of the kidney? Upon this question, Cases 3, 4, 5, 6, and 7, tend to throw considerable light.

"To begin, then, with Case 3;—and let us, first of all, endeavour to reconcile it with the former hypothesis. The hypertrophy of the left ventricle, then, was notable, and its injecting force must have been excessive: there is, however, much difficulty in accounting for this hypertrophy, as there was no valvular disease; unless we refer it to the morbid changes which had taken place in the arteries, and which may certainly be regarded as a real and a sufficient cause for the phænomenon: for it must be obvious that the disease of the arteries was not produced by the violence which might have been done to their lining membranes by the too-powerful ventricles; since, in the arch of the aorta, where such violence would have been the greatest, the injury was less than in the descending portion, where the force of the heart's action must have been far less.

"According to this hypothesis, then, the arterial derangement must have been the primary lesion; which, by the impediment it offered to the free passage of the blood, gave rise to hypertrophy of the left ventricle, which, in its turn, by its too-powerful injecting force, caused a congestion of the kidneys, which led to their ultimate disorganization. It need hardly be remarked, that there is a contradiction involved in the very terms of the explanation: but further than this there is the difficulty of explaining why the kidneys should have suffered so much, whilst the liver, which is generally the first organ to be affected by disease of the heart, should have escaped; and, also, of accounting for disease of the arteries, which there is every reason to believe is a rare affection, when occurring idiopathically.

"Let us now assume, that the disease of the kidney was the primary affection: and in so doing, we may observe that we are not met *in limine* by the same difficulty which we had to encounter on the former hypothesis; for although to assume that the kidneys are often primarily affected with this disease would be, in some measure, begging the question, yet numberless analogies may be drawn from other secreting organs, to shew that over-stimulation may give rise to hyperæmia and subsequent disorganization: and that the kidneys were so stimulated in this case, is rendered highly probable, from the history of the invasion of the disease, as well as the previous habits of the patient. It is also ascertained that changes were produced in the condition both of the secretion of the kidneys and of the blood; the former being deficient in some of its natural solid contents, which were found to exist in excess in the blood; whilst the latter fluid was deficient in an important solid ingredient, which was being continually abstracted by the kidneys; so that there was a two-fold change produced in the latter fluid; namely, the presence of an irritating substance, which it is the office of the kidneys in health to remove; and also the absence of a substance, which, independently of other important purposes in the animal economy, gives to the blood a viscidity which (according to the observations of Magendie) renders it more easy of transmission through the capillary vessels. We have, then, a cause sufficient to account for the disease in the arterial tunics, whereby the circulation in the larger vessels must be impeded; as well as a further obstacle to the transmission of the blood, resulting from the change in the blood itself: both of which circumstances must have co-operated in producing the hypertrophy of the left ventricle. It cannot then, I think, be doubted, that the latter hypothesis accounts for the phenomena in this instance more readily than the former.

"The same line of reasoning, with very little alteration, applies to Cases 4 and 5: though perhaps, in the latter, there might be some suspicion of disease in the chest giving rise to venous congestion in some of the abdominal viscera, and, amongst others, in the kidneys, although not to a sufficient extent to account for the change which those organs had undergone.

"In Case 4, indeed, there had been cough and dyspnœa for some time, before any dropsical swelling manifested itself: but although it is by no means improbable, from the history of the case, that bronchitis existed, yet it is now so well ascertained that renal disease may exist without any dropsical swelling, and also that, when it does exist, it is so uncertain in its situation, that we are not justified by its absence in assuming that the kidneys were sound, or even that disease in those organs might not have existed to an extent capable of producing œdema of the lungs: whereas the copious diuresis which existed for some time previously would be sufficient to lead us to believe that the kidneys had been for a long time in a state of over-activity—a belief which is most fully confirmed by the advanced state of disorganization in which these organs were found after death.

"In Case 5, again, we have evidence of a still greater amount of obstructive disease in the lungs and heart, though the left side of the latter organ was principally affected; so that, although there existed a cause for venous congestion in the abdominal viscera, and, amongst the rest, in the kidneys, it was not sufficient to account for the extent of degeneration which these organs had undergone.

"In Case 6, again, we have a still greater amount of obstruction to the return of the venous blood: and, with it, considerable disease of the kidney, which, however, was principally of a congestive character. But it is in Case 7 that we find the causes of venous obstruction in their fullest extent; there being great dilatation and hypertrophy of the right ventricle, owing to the impediment to the pulmonary circulation, caused by the narrowing of the left auriculo-ventricular opening on the right side of the heart: yet here the disease in the kidney was less advanced than in any of the preceding cases, but that disease was of a congestive character, and during life the urine contained a scarcely appreciable quantity of albumen.

"The five last cases, in fact, present a series in which the renal disease becomes less and less, whilst the thoracic disease becomes greater and greater: the former affection, however, assuming more of the congestive character, as we approach the close of the series.

"We are then, I think, justified in concluding, that although disease in the heart or lungs may, by the impediment which it causes to the return of blood through the veins, give rise to congestion in the kidneys, in common with other organs in the abdomen, and thereby derange their functions, and in some cases lead to their ultimate disorganization, yet, as such disease was the least in those cases where the renal disease was the most advanced, and the converse, we are not to conclude that such disease in the chest is the universal or even frequent cause of the true Renal Disease; and that we must look elsewhere for the seat of the primary lesion in this formidable malady."

This reasoning is ingenious, if not conclusive, and the subject is one that merits close attention. The remaining Cases in the Report will repay a careful perusal. We see little more to notice.

---

### ST. GEORGE'S HOSPITAL.

### REPORT OF SOME CASES OCCURRING UNDER THE CARE OF MR. HENRY JAMES JOHNSON, ASSISTANT-SURGEON TO THE HOSPITAL.

Perhaps some apology is due for obtruding on the notice of the reader cases of so desultory, if not trivial a character as the succeeding. But as life is made up of small incidents, and the most happy and successful man is he who contrives to extract pleasure and profit from them, so professional existence is mainly composed of little matters, which, managed well or ill, constitute the difference between satisfaction and discomfort, perhaps between fortune and failure.

Even in a great Metropolitan Hospital, with all its appliances for attracting to itself what is rare and severe in the mass of human maladies, the bulk of the cases are of common occurrence, and of a mild description. They therefore present those opportunities for observation, practice, and experience, best calculated to qualify the physician or the surgeon for the duties of every day. To neglect them is to neglect the very business of life; to study them, goes far towards making him master of it. It is a trite observation, that the student too frequently *does* neglect them, and the practitioner too late regrets it.

I have always been of opinion, that it is the *duty* of those who have the charge of Hospitals, to render the facts that they contain as available as possible for the whole profession. These appointments are necessarily few, and their fortunate possessors should look on them not as prizes in a lottery, given for the exclusive advantage of the drawers, but as public trusts for the public benefit. Actuated, then, by this conviction, I have felt that even ordinary cases might convey some information to those whose means of obtaining it are straightened, and tend to bring the isolated country surgeon to the level of the knowledge of the capital. For, in hospitals, new medicines can be given, new methods can be tried, to an extent not permitted by the narrow limits, and strict responsibilities of private practice.

Whatever may be thought of the performance of the task, I trust that no exception will be taken to the spirit of it. I am sensible that, at present, the execution lags sadly behind the promise, but venture to indulge the hope that something more worthy may be effected bye-and-bye.

The first subject to which I shall allude is the—

### I. MANAGEMENT OF ULCERS OF THE LEGS.

Those who have been much about Dispensaries or Hospitals are aware both of the frequency and the obstinacy of ulcers of the lower limbs in the out-door patients. They often tire out surgeon, dresser, and all, and week after week, and month after month, there is the same sore with the surface just as foul, or the edges just as hard as ever, to the shame and confusion of ointments of all colours and bandaging of the first order. Any treatment which has some success to boast of, and curtails the duration of these opprobia *chirurgorum*, is "a consummation devoutly to be wished for."

On first assuming the office, I was naturally inclined, "stare super antiquas vias," and to appeal to poultices, unguents, or strapping as the fit and proper things for ulcerated legs. But in spite of them, the dresser, and myself, too many of those legs grew little better, and some got even worse. It was obvious that either a happier method must be found, or else that sore legs were a nuisance not likely to be materially abated. A plan was tried by the Assistant House-Surgeon, and myself, which, on the whole, has not disappointed our anticipations, and has really effected so much benefit, as to render an ulcer of the lower limbs a hopeful sort of case. Whatever claims others may possess to the integral parts of the plan in question, I beg to make freely over to them.

I may be permitted to state that these observations are neither calculated nor intended for a copious account of the varieties or treatment of ulcers. They are hints for their management, and nothing more. But to the point.

As a general rule, subject unquestionably to many exceptions, moist applications seem to suit ulcers best—a piece of lint dipped in the liquid, whatever it may be, larger than the sore on which it is laid, and covered with a piece of fine oiled silk, larger still, is the sort of dressing employed. The lint should be changed sufficiently often, or, at all events, dipped frequently enough in the liquid, to ensure its permanent moisture. Three or four such applications in the twenty-four hours are, in most instances, sufficient. When the discharge is copious, attention to this is particularly necessary. A girl with an ulcer on the arm, of large dimensions, the consequence of a burn, allowed the lint to remain in contact with it for more than forty-eight hours. The matter had collected and became offensive, and the granulations were absorbed, and the sore much deepened.

In most cases, it is well to employ a bandage. If the patient maintains the horizontal posture, this is not so requisite. The bandage should, in general, be applied lightly, and may, in some instances, be advantageously kept wet.

It is foreign to my purpose to inquire into the manner in which these liquid dressings act. It is probable, however, that the sustained temperature of the sore and its vicinity occasioned by the prevention of evaporation, operates as a stimulus, while the moistened lint forms a sort of nidus for the granulations. That the temperature of the part is kept up is certain.

I have, hitherto, said nothing of the nature of the liquid selected as an application. My impression is, that in the bulk of cases some gentle stimulus is best. I have tried plain water, the solution of the nitrate of silver, of the sulphate of zinc, of the soda chlorinata, of opium, and the black wash. Amongst all these, the solution of the sulphate of zinc, in the proportion of two grains to the ounce of water, has appeared to suit most generally. When the sore has been more than usually painful, the solution of opium, of a similar strength, has agreed better. And foul, dirty, cachectic looking ulcers have seemed most benefited by the solution of the soda chlorinata, one part of which is dissolved in sixteen of water. These, however, are merely hints thrown out, as approximations to the truth. It remains for other surgeons, and for further experiments, to determine what may be their value.

I have observed that a light bandage is usually adviseable. In some cases, more decided support is needed. Where the ulcer depends on a varicose state of the veins of the limb, without that inflamed, and eczematous or psoriasitic state of skin to which varices of the veins often lead, soap-strapping may be usefully employed. Perhaps the best method of applying this consists in setting on the strips to just *below* the ulcer, *above* which other strips commence, the latter being the looser. The ulcer itself is thus left bare for the application of the dressing, over which farther strips are daily placed. The advantage of this is, that the ulcer can be got at without disturbing the greater portion of the strapping.

I know not what will be said by some gentlemen, when I own to the weakness of at times resorting to a poultice. Yet it has appeared occasionally to give relief, even when water-dressing has failed. When the sore is irritable and inflamed, one of bread and water, or of bread and Goulard water, laid on at night with a piece of oiled skin over it, has been productive of much comfort. At the same time, it is only fair to admit, that poultices may be grossly abused, and as ordinary applications are useless, or worse. When the sore is sloughy and inclined to phagedæna the opium poultice is invaluable.

In that inflamed condition of skin to which allusion has been made, goulard water applied through the medium of rags, or a bandage covered with oiled skin, commonly gives much relief. It does not seem to agree so well with the ulcer itself, which may be dressed in the ordinary way. It is needless to observe, that for cases such as these, rest is particularly requisite.

Such are the few observations I would make upon the local treatment. Possibly a few may also be permitted on the general.

Of course it is requisite to attend to the state of the secretions. If the stomach, the liver, the kidneys, should be out of order, such medicines should be given as are calculated to restore their functions. It would be impertinent to dwell on *them*.

Of the value of purging there can be little doubt. Where the tongue is loaded and foul, with but moderate or impaired constitutional power, a condition very common amongst out-patients, bitter purgatives agree. The infusion of gentian with the tincture, and some sulphate and carbonate of magnesia, form a good though nasty compound. In females, with defective menstruation, or, at a later period with impaired digestive powers and low tone, the compound decoction of aloes, with or without senna, is useful.

Tonics are, in most cases, required after purgatives or with them. Circumstances must determine their selection. In cachexia, mercurial or otherwise, sarsaparilla bears the bell. Quina, gentian, steel have their several applications. A cheap and a good tonic in persons with a weak circulation, and not disposed to cerebral congestion, is the compound steel pill. It is a great favourite with my excellent colleague, Mr. Cutler.

But there are very many cases in which the compound soap pill acts admirably. Elderly persons, with a varicose condition of the veins of the lower limbs, a languid pulse, and a feeble and not excitable habit, are sometimes wonderfully bettered by it. Nor does it appear to affect the head or constipate the bowels, so much as might have been anticipated. The dose may be three grains twice or thrice daily, and senna or aloes may be taken in the morning for the bowels' sake.

I am not so sure that this medicine suits the young or middled aged—undoubtedly it does not suit *so* well. It is apt to distress the head. In one woman between 20 and 30 years of age, disposed to flushed face and to cerebral fulness, it aggravated the latter to such a degree as to compel its discontinuance. In the cases I have selected for it, it is a valuable remedy.

With these observations, I will now take the liberty of giving the brief particulars of two or three cases intended to illustrate them. They have been drawn up by my brother, Mr. Athol Johnson, who has acted as Clinical Clerk. They are mostly in the condensed form of Report, and have been selected merely as samples, from amongst a considerable number.

CASE 1.—*Old Varicose Ulcer—Comp. Soap Pill and Lotio Zinci Sulphatis—Cure.*—Hannah Sadgrove, æt. 27, applied at St. George's Hospital, on the 18th of January, on account of a large varicose ulcer of the leg of long standing; a variety of medicines were administered, and poultices, ointments, &c. applied, without any benefit. On the 28th March, the notes state that there was "no improvement: sores sloughy." Three grains of the compound soap pill were ordered to be taken three times a day, and the lotio sod. chlor. and afterwards the lotio zinci sulph. applied locally. On the 19th of April, the patient was discharged, cured, the ulcers being firmly healed.

CASE 2.—*Cachectic Ulcers of Leg—Comp. Soap Pill and Sol. Arg. Nit.—Cure.*

Catherine Bennett, æt. 30, servant. This patient applied at the Hospital on the 1st of Feb. with a cachectic ulcer of the leg, about the size of a crown-piece, which had first appeared about three months before; the veins of the extremities were also considerably enlarged. She was placed upon steel, with occasional purgatives, and a variety of applications, ointments, poultices, &c. were tried, without any apparent benefit. On March 25th three grains of the compound soap-pill were ordered to be taken daily, and a weak solution of nitrate of silver applied, under oiled silk, to the leg. The beneficial effect was immediate; the sore assumed a healthy character, which it never subsequently lost, and on April 23, the patient was discharged, cured, the ulcer having been for some time soundly healed.

CASE 3.—*Old Sloughy Ulcer—Comp. Soap Pill and Sol. Sod. Chlor.—Cure.*

Henry Butterfield, æt. 48, made O. P. Feb. 1st, 1843.—This was one of the worst cases of old sloughy ulcer, which had been seen at the Hospital for some time. It was of very large size, and there was great induration of the cellular membrane of the whole leg, with an extremely fætid discharge. The compound steel pill and other tonics were employed without any benefit. On the 4th April, there being then no improvement in the state of the ulcer, three grains of the compound soap pill were ordered to be taken three times a day, a warm bath twice a week, and a lotion composed of one part of liq. sod. chlor. to fifteen of water, applied under oiled silk. From this time a gradual improvement took place, and on May 16th he was discharged, the ulcer being quite healed, the cicatrix appearing good and healthy, and the induration nearly all removed.

CASE 4.—*Very foul Varicose Ulcer—Gentian Mixture, and Sulph. Zinc Lotion—Cure.*

Mary Smith, æt. 47, washerwoman. Applied on the 12th April, with a painful varicose ulcer on the leg, of about the size of a shilling, with dark-coloured edges, and excavated surface, discharging foul and fætid matter. The health was much impaired.

She was directed to take gentian with carbonate and sulphate of magnesia twice a day, and to apply the sulphate of zinc lotion (two grains to the ounce) under oiled silk. The sore immediately improved in appearance, and by the 18th May was quite healed. She was subsequently attacked with pain in the head, rendering it necessary to cup her, but the ulcer remained healed.

CASE 5.—*Old Varicose Ulcers—Gentian Mixture and Opiate Lotion—Cure.*

Harriet Pukett, æt. 40. This woman had been suffering from very large varicose ulcers of the legs for a long time: when she applied for relief at the Hospital, about the beginning of February, the ulcers were in an indolent condition, and had then existed for about eight months; the health was also considerably impaired. Bitter purges, tonics, &c. were tried, and a variety of ointments applied to the sores, without much benefit to the ulcers, the report on the 28th March being that "the granulations were livid and flabby." The compound soap pill was then ordered, but this affected the head so much that it was necessary to leave it off. Gentian with carb. magnes. and sulph. mag. was then tried, with the sulphate of zinc lotion, and afterwards Goulard water, both these applications, however, gave so much pain that they were omitted, and on April 23, a solution of opium, (gr. ij. ad ℥j.) was substituted. On the 4th of May, the ulcers were all healed.

Case 6.—*Cachectic Ulcerations of both Legs, of long standing—Tonics, Black-wash and Sol. Sod. Chlor.—Cure.*

Benjamin Bennett, æt. 42, applied at the Hospital on the 26th April, with cachectic ulcers on the front of either leg, some of which were as large as a crown-piece. There were also scars on the forehead, &c. of other cachectic sores, and the remains of destructive ulceration of the soft palate.

Three years before this he had a sore on the penis, for which he was freely salivated; about a year afterwards he was attacked with sore-throat, &c. which had continued, off and on, up to the time of his application at the Hospital.

He was ordered to take the haustus cinchonæ, with pot. iod. gr. v, twice a day, the bowels were kept moderately open, and the lotio nigra applied to one leg, the sol. sod. chlor. to the other, under oiled silk. On the 2nd May, the granulations were florid, in some ulcers even exuberant. There was no great difference in the progress of the two legs. The granulations were kept down by means of the argenti nitras, and on the 1st June, the patient ceased to attend, the sores on both legs being quite healed.

### II. Incision of Bursæ containing the "Hydatidiform," or "Melon-seed" Bodies.

Every surgeon is aware that both the simple and vaginiform bursæ, but more especially the latter, are liable to be distended not only with an increase of their natural secretion, but also with numerous small and separate bodies, oval or rounded, and frequently bearing a close resemblance in their form to melon-seeds. On the Continent, they have been considered as allied in nature to hydatids, but the prevalent, and more probable opinion, in this country, regards them as merely lymph broken down and rounded by attrition in a fluid medium. One of the cases, which will be detailed, appears to lend a strong confirmation to that idea.

My object, however, in touching on the subject, is rather connected with the treatment of the disease, and the propriety of resorting to the knife.

When the bursa is simply filled with fluid, with or without thickening of its parietes, blistering and pressure are commonly effective in reducing the enlargement. The same means, no doubt, may frequently succeed in procuring the absorption of effused lymph. But when this is disintegrated, and the "melon-seed" fragments have been formed, it is questionable, alike in theory and fact, whether any applications on the surface can exert much influence on them. Now, as bursæ in this state occasion a good deal of annoyance to the patient, it becomes a question whether an incision with the view to the removal of the bodies can be recommended.

Although it *has* been recommended and performed, there have occurred so many instances of subsequent inflammation and suppuration, that the experience of surgeons is against the step.

It is fair to suppose that the danger is this:—a cavity is opened, which is prone by the nature of its tissue (a synovial one) to suppurative action—pus is secreted—it gets no free vent—and hence the mischief. If this be so, it follows that the more complex and ramified the sac, the more imminent the risk. The sub-cutaneous simple bursæ, such as that above the patella, would be favourably placed for an incision—the deeper simple bursæ, or the vaginiform, would be unpropitiously circumstanced. And such, I should say, is the fact. The supra-patellar or olecranal bursa may be laid open, with comparative, though not always, absolute impunity—the scapular, trochanteric, carpal and tibio-tarsal bursæ may not.

The risks, in fact, that wait on meddling with these latter, are great. In two instances, I have witnessed extensive suppuration within it and around it, confinement of matter, extreme constitutional irritation, and fatal secondary inflammation from making an opening into the bursa, enlarged and filled with fluid, over the inferior angle of the scapula. In three instances, I have seen wide-spread suppuration in the fore-arm, from incision of the vaginiform bursa in the wrist, connected with the flexor tendons. We had lately a patient in the hospital, in whom this bursa was much enlarged, and presented a swelling in the palm, communicating under the transverse carpal ligament, with another in the lower part of the fore-arm. Fluid could be pressed from one swelling to the other, and, I think, there was the crepitation indicative of lymph. An incision was made into the palmar swelling—much inflammation and suppuration in the fore-arm followed, and the patient, of her own will, quitted the hospital, in a very dangerous state. I have not heard the result.

But though, as a general rule, it is inadviseable to make an opening into the vaginiform bursæ, and the deeper-seated simple ones, it would be going too far to prohibit such a measure altogether. The inconvenience may be such as to justify the encounter of some risk—or suppuration may be actually occurring or established in the cavity. Besides, it may happen that the bursa has been incautiously punctured, mistaken, perhaps, for something else, or supposed to contain only fluid, when the "melon-seed" bodies, &c. are within it. It becomes then, a desideratum to determine, if a puncture is to be made, what sort of a puncture that should be, and what steps are to be taken to obviate the dangers which *any* puncture may give rise to.

It appears to me that, if we do make an opening, it should be a free one—an opening which may not only discharge the actual contents of the bag, but render confinement of the pus that is to form impossible. No half-measure can be safe—lodgment of matter must be prevented, not relieved. These sacs are as much disposed to form lymph as pus, and a moderate opening will be partially, at all events, hemmed round with the former. Whatever we may do afterwards will probably not retrieve this first false-step—some purulent accumulation will take place—and if it does so, the risk of secondary inflammations is imminent.

The following case may seem to support these observations. I would remark that the diagnosis, prior to the puncture, was uncertain. The gentleman, under whose care the patient was, previously to his application to me, conceived that the case was one of fatty tumor. The puncture with the exploring-needle determined of course the existence of fluid, but its clearness and facility of exit deceived me, and led me to imagine that it exclusively occupied the sac. This induced me to make the subsequent puncture more readily, and I experienced some surprise when the quantity of liquid proved not to exceed two or three drachms, the bulk of the contents being solid. However, the opening *was* made, and immediately, in accordance with the principles that have been stated, I enlarged the incision to fully two inches, and introduced between its edges such a dossil of wet lint as kept them effectually asunder. The result appears to have stamped the propriety of the practice.

There is little doubt that the bursa was that connected with the tendon of the glutæus maximus. This stretches beneath the fascia lata. It was in this instance of large dimensions, nearly capable of holding a fist, and when the masses of lymph were made to tumble out of it, the look of the thing was unpleasant. I am confident that, had matter once been confined there, the life of the patient would have been in great hazard.

The process of transformation of amorphous masses of gelatinous-like lymph into the "melon-seed" bodies, was equally striking and obvious. The successive stages were apparent in the different portions. I now subjoin the case.

CASE 7.—Wm. Weare, æt. 25, servant. Admitted April 19th, 1843, with a globular swelling about as large as a moderate-sized melon, superficially subcutaneous, but stretching deep, situated on the right buttock, above and behind the trochanter major. No distinct fluctuation, but feels rather like a fatty tumor. On bending the thigh, it becomes much more tense, prominent, and defined, and the fluctuation is much more evident. Skin over it unaltered; no pain.

States that he first discovered the swelling, accidentally, five months ago; it was then of about the diameter of a crown-piece, and has gradually increased in size ever since. Knows of no cause.

Has been attending as out-patient for five or six weeks, when the tumor was punctured with a grooved needle, and some yellowish clear fluid escaped.

21st. Punctured with lancet. Two or three drachms only of fluid escaped, the remainder of the contents consisting of the gelatinous sort of matter which is found in bursæ and vaginiform sheaths. Masses of considerable size, and irregular form; one, opaque and somewhat broken up, was apparently in process of transformation into "melon-seed" bodies. It was necessary to squeeze the cyst in order to get out the masses, some of which were left behind, and the opening was enlarged to 1½ or 2 inches; a piece of wet lint was then introduced into the dependent extremity of the wound.

Catap. panis calid. H. sennæ.

22d. No symptoms of irritation. Edges already granulating. Sac nearly empty. Some yellow opaque masses were squeezed out, consisting of portions which, with very little separation and attrition, would be "melon-seed bodies" or "hydatids." *Vespere.* Heat of skin, with foul tongue, and some pain.

H. salin. efferv. 4tis horis.

25th. Has continued to have some little feverishness. There has been copious discharge, generally of thin yellow matter, but now rather thicker. Pain at times. No surrounding inflammation. Tongue still rather foul.

Pil. hydrarg.
Ext. coloc. comp. āā gr. v. h. n.
Haustus sennæ cras mane.
Haustus salinus bis in die.

May 2nd. Complains of severe pain across the forehead. Tongue still foul.

Infus. ros. ℥iss.

Magnes. sulph. ʒj.

Acid. sulph. dil. ♏x. bis in die.

Omitt. alia. Ordinary diet.

8th. Has lost the pain in the head. A little purulent discharge from the wound which is contracting. Health good.

24th. Wound now healed to a mere spot, furnishing only a little oozing. Discharged.

